# Classification of human chronotype based on fMRI network-based statistics

**DOI:** 10.3389/fnins.2023.1147219

**Published:** 2023-06-05

**Authors:** Sophie L. Mason, Leandro Junges, Wessel Woldman, Elise R. Facer-Childs, Brunno M. de Campos, Andrew P. Bagshaw, John R. Terry

**Affiliations:** ^1^School of Mathematics, College of Engineering and Physical Sciences, University of Birmingham, Birmingham, United Kingdom; ^2^Centre for Systems Modelling and Quantitative Biomedicine, University of Birmingham, Birmingham, United Kingdom; ^3^Turner Institute for Brain and Mental Health, School of Psychological Sciences, Monash University, Clayton, VIC, Australia; ^4^Danny Frawley Centre for Health and Wellbeing, Melbourne, VIC, Australia; ^5^Centre for Human Brain Health, College of Life and Environmental Sciences, University of Birmingham, Birmingham, United Kingdom; ^6^Faculty of Health and Medical Sciences, University of Surrey, Surrey, United Kingdom; ^7^Department of Neurology, University of Campinas, São Paulo, Brazil

**Keywords:** chronotype (morningness-eveningness), functional connectivity, fMRI, classifier, network-based statistical (NBS) analysis, functional networks, graph theory

## Abstract

Chronotype—the relationship between the internal circadian physiology of an individual and the external 24-h light-dark cycle—is increasingly implicated in mental health and cognition. Individuals presenting with a late chronotype have an increased likelihood of developing depression, and can display reduced cognitive performance during the societal 9–5 day. However, the interplay between physiological rhythms and the brain networks that underpin cognition and mental health is not well-understood. To address this issue, we use rs-fMRI collected from 16 people with an early chronotype and 22 people with a late chronotype over three scanning sessions. We develop a classification framework utilizing the Network Based-Statistic methodology, to understand if differentiable information about chronotype is embedded in functional brain networks and how this changes throughout the day. We find evidence of subnetworks throughout the day that differ between extreme chronotypes such that high accuracy can occur, describe rigorous threshold criteria for achieving 97.3% accuracy in the Evening and investigate how the same conditions hinder accuracy for other scanning sessions. Revealing differences in functional brain networks based on extreme chronotype suggests future avenues of research that may ultimately better characterize the relationship between internal physiology, external perturbations, brain networks, and disease.

## 1. Introduction

Almost all bodily functions depend fundamentally on oscillations. A diversity of biological clocks tightly interconnect to control processes over time scales of hours such as sleep stages or body temperature through to days and months, for example, hormone release or the female menstrual cycle (Kondratova and Kondratov, 2012).

Processes that oscillate with a period of around a day are called *circadian*. These include neurobehavioral (i.e., attention or mood), hormonal (i.e., melatonin or cortisol secretion) and physiological (i.e., heart rate or body temperature) (Wirz-Justice, 2007). Circadian rhythms in the context of sleep refer to the naturally occurring oscillatory nature of a human's high and low sleep propensity (Borbély and Achermann, 1999). However, the phase relationship between the internal circadian rhythm and the external clock time can differ markedly between individuals, contributing to the classification of people according to their chronotypes (Blautzik et al., 2013). Chronotype classification will fall across a spectrum, with early circadian phenotypes (ECPs) and late circadian phenotypes (LCPs) sitting at the two extremes. These two extreme phenotypes are often colloquially referred to as “Morning Larks” and “Night Owls”. Upon removing external social obligations, the sleep of ECPs and LCPs will show little difference—except for the phase between circadian rhythm and clock time. For example, driven by the endogenous circadian rhythm of the individual, extreme ECPs may wake up at the same time that extreme LCPs are falling to sleep (Roenneberg et al., 2003).

The Munich Chronotype Questionnaire (MCTQ), first introduced in Roenneberg et al. (2003), is a standard tool for chronotype classification alongside actigraphy data and finding peaks in cortisol and melatonin concentrations from saliva samples (Facer-Childs, 2018). These methods permit classification based upon physiological markers; however, they do not provide an insight into the underlying processes that lead to circadian rhythmicity and how these oscillations ultimately impact upon health and brain function.

Chronotype is a known risk factor for a variety of common health conditions. In particular, late chronotypes are associated with an increased risk of cancer, type 2 diabetes, as well as increased BMI and obesity (Hug et al., 2019). Further, late chronotype is a known risk factor for the development of depression (Levandovski et al., 2011) as well as being predictive of variability in cognitive outcomes across the day (Facer-Childs, 2018). The role of functional brain networks in supporting healthy brain function and the disruptions that lead to impaired performance and disease are increasingly understood in many fields such as depression (Liu et al., 2020) as well as cognition both in terms of cognitive architecture (Petersen and Sporns, 2015) as well as cognitive aging (Terry et al., 2004; Hausman et al., 2020). Consequently, an increased understanding of the relationship between chronotype and functional brain networks could provide insight into the increased risk of mental health and cognitive outcomes, the reason for the disparity of such impacts along the chronotype spectrum, and therefore areas upon which to focus future research. In addition, a greater understanding of how chronotype impacts the brain's functional network could provide support for practical changes in society, such as the school day starting later for adolescent children (Adolescent Sleep Working Group et al., 2014) or greater flexibility in workplace hours (Vetter et al., 2015).

A common technique to investigate how macroscale neurological processes occur or manifest in the brain is fMRI (functional Magnetic Resonance Imaging). FMRI produces a blood-oxygen-level-dependent timeseries for each voxel—the 3D analog of a pixel—in the brain. Groups of spatially and functionally related voxels can then be grouped together into regions of interest (ROIs), with each ROI having an averaged timeseries of the voxels within. Functional connectivity (FC)—the statistical dependencies between time-series (Friston, 2011)—can then be calculated between pairs of voxels, pairs of ROIs or between ROIs and other voxels. A seed-based approach focuses on the statistical dependencies between a specific voxel or ROI, known as the seed, and all other voxels. In a connectivity-based approach the entire brain network is represented as a functional network (FN) with ROIs as nodes and connections (edges) between them determined by FC. Creating a FN permits tools and techniques from mathematics, such as graph theory and the calculation of graph metrics (Liu et al., 2014; Farahani et al., 2021) to be utilized.

Motivated by the success of seed-based and connectivity-based pipelines within areas such as time of day effects (Hodkinson et al., 2014), as well as chronic acute (Farahani et al., 2019) and prolonged sleep deprivation (Liu et al., 2014), some limited studies exploring the role of chronobiology on functional networks have been undertaken. For example, Facer-Childs et al. (2019) seeded in the medial Prefrontal Cortex and Posterior Cingulate Cortex within the Default Mode Network. For both seeds, using the contrast ECP > LCP, the respective clusters were predictive of attentional performance as measured through a psychomotor vigilance test. In addition, resting state FC recorded from the medial Prefrontal Cortex was predictive of Stroop performance, and the Posterior Cingulate Cortex was predictive of subjective sleepiness measured using the Karolinska Sleepiness Scale (KSS). Facer-Childs et al. (2021) also completed a similar analysis within the Motor Network system, where Motor Network resting state FC was shown to contribute to individual variability in motor performance. On the other hand, Fafrowicz et al. (2019) seeded in 36 regions throughout the brain. Correlation of the resulting clusters' FC to time of day, chronotype, and time of day × chronotype was considered. The effect of chronotype manifested itself differently in these studies: chronotype alongside rs-fMRI was successfully used as a predictor of cognition (Facer-Childs et al., 2019), whereas Fafrowicz et al. (2019) failed to find a significant difference between extreme chronotypes with rs-fMRI and instead only found significant time of day effects.

On the other hand, Farahani et al. (2021, 2022) chose a more traditional connectivity-based approach of creating individual FNs before binarizing the networks at a range of density-based thresholding values. Graph metrics were then calculated using the binarized networks before being considered for group-level differences between ECPs and LCPs in the morning and evening. In these works, a total of 15 graph metrics were calculated ranging from local scale to mesoscale and global scale. In all cases, graph metrics did not significantly differ based upon chronotype. However, significant time of day effects were once again seen when comparing graph metrics associated with different scans in the morning and evening for the small-world index, assortativity and network synchronization and within specific nodes when calculating degree centrality and betweenness centrality. Highlighting the lack of success in differentiating ECPs and LCPs within current connectivity-based pipelines.

The limited research and conflicting findings on the role of chronotype on functional networks derived from fMRI data is suggestive of chronotype having relatively subtle effects on functional brain networks, that cannot easily be detected using traditional seed or connectivity-based pipelines. An alternative approach to creating a classifier based on the graph metrics of FNs is utilizing Network-Based Statistic (NBS). NBS is a connectivity-based method, that aims to find a subnetwork consisting of edges showing high differentiation levels between the functional networks of two groups (typically a control and patient cohort)—called a *dysconnected network*. The term dysconnected network is not to be confused with *disconnected network* a word commonly used within graph theory to refer to a network in which two nodes have no path between them. NBS is neither a seed-based method nor a connectivity-based method, but an intermediate. For example, NBS does not require a seed like seed-based approaches, neither is it used to calculate graph metrics per subject like many connectivity-based approaches. However, there are similarities between NBS and these methods. For instance, NBS utilizes connectivity-based approaches both through the method requiring a FN for each participant and the method producing a single group-level FN—the dysconnected network. Also, like seed-based approaches NBS utilizes contrasts and statistical tests to understand directed group-level differences—in this case the differences in the edge weights between two ROIs rather than two voxels. Therefore, NBS encompasses a mixture of the two methods whilst also being distinctly different.

The original application of NBS explored differences between the functional networks of people with schizophrenia and controls (Zalesky et al., 2010) (RRID:SCR_002454). Since then, NBS has been explored in a range of neurological contexts from the effects of habitual coffee drinking on the FC patterns in the brain (Magalhães et al., 2021) to considering how age and intelligence (DeSerisy et al., 2021) or symptoms of ADHD (Cocchi et al., 2012) can be correlated with the FC in dysconnected edges found from NBS. A recent extension of NBS (NBS-Predict) has also been published (Serin et al., 2021). It uses a training set to select relevant features from NBS networks for the classification of test subjects. In contrast, our classifier evaluates whether an ECP or LCP label of the test subject leads to greater differentiation between the two groups in a group-level comparison. The use of the test subject therefore differs between the two classification approaches.

The methodology underpinning the NBS approach is described in detail in Zalesky et al. (2010) and is published alongside a freely available NBS toolbox for MATLAB (MathWorks, USA). Alongside the recent extension NBS-Predict for the classification of test subjects (Serin et al., 2021). For completeness, a brief overview of NBS in the context of chronotype is provided below.

To consider whether NBS may be better suited than seed-based or graph metric connectivity-based pipelines for differentiation between ECPs and LCPs we introduce a classifier to assess the ability of NBS-derived dysconnected networks for classifying individuals as ECP, LCP, or unclear. Within NBS, a t-statistic thresholding step is required. Therefore, we originally create a classifier, with an objective method for selecting this threshold. The t-statistic threshold selection criteria is based on the assumption of connectedness for the undirected dysconnected networks. In particular, t-statistic thresholds which create minimum connected components (MCCs) are utilized, therefore ensuring all ROIs are present with an undetermined number of edges. With no prior knowledge of the subjects this accounts for the potential uniqueness in an individual's subnetwork of importance by assuming all ROIs provide information and must therefore form part of the subnetwork.

Broadly, the classifier uses NBS to create and compare the dysconnected networks resulting from labeling a test subject as first an ECP and then an LCP. We compare the significance of these dysconnected networks at specific t-statistic thresholds, alongside their size (number of edges), to classify the test subject—significance and larger networks are taken as an indication of correct labeling.

Additional analyses were performed using the classifier within a classification framework to examine time of day effects. In addition, the stability of the classifier on subsets of the dataset created from leaving out one subject at a time was considered leading to an investigation into the reasons why the removal of certain subjects resulted in large changes in accuracy and possible ways to mitigate this effect.

Overall, chronotype can greatly affect both the day-to-day life as well as the long term physical and mental health of individuals. However, little information is known about the effect of chronotype on rs-FNs. In this work, we use a novel approach of a classification framework based on NBS to understand if differential information between extreme chronotypes is present in rs-fMRI data. Data from three scanning sessions across the day are considered; therefore the ability of the NBS classifier to differentiate ECPs and LCPs at different times of the day will also be assessed as it is unlikely the rs-FNs of the two groups will differ in the same manner across the entire day. Given the lack of significant differences in the graph metrics (Farahani et al., 2021, 2022) and seed-based functional connectivity (Fafrowicz et al., 2019) of ECPs and LCPs in current literature, finding scenarios of clear differentiation between groups would provide evidence that chronotype can affect rs-FNs and motivate future studies.

## 2. Materials and methods

The chronotype dataset analyzed in this study was first presented by Facer-Childs et al. (2019), which can be referred to for a detailed description of the data acquisition and preprocessing methodology. Ethical approval was provided by the Research Ethics Committee at the University of Birmingham, before any data acquisition started. Participants had at least 48 h before the start of the study to read over information sheets and consent forms, and they were free to end their participation at any time. In addition, the University of Birmingham's Advisory Group on the Control of Biological Hazards approved the COSHH risk assessments and biological assessment forms that were completed. All the data and samples were given by participants voluntarily and were fully anonymized.

### 2.1. Participants

Thirty eight participants were enrolled into the study, with an average age of 22.7 ± 4.2 years (mean ± standard deviation), of whom 24 were female. Exclusion criteria included (1) prior diagnosis of any sleep, neurological, or psychiatric disorders (2) use of medication that would knowingly affect sleep (3) an intermediate chronotype classification from the MCTQ. For 2 weeks prior to the scanning sessions, participants were instructed to follow their preferred sleep pattern, with no restrictions imposed by the study except for 2 h before the scanning sessions when alcohol and caffeine consumption, as well as exercise, were prohibited.

16 subjects were classified as ECPs (age 24.7 ± 4.6 years, 9 females) and 22 as LCPs (age 21.3 ± 3.3 years, 15 females). Chronotypes were determined by the outcome of the MCTQ and were further validated by the analysis of Dim Light Melatonin Onset (DLMO) and Cortisol Awakening Response (CAR) times as well as sleep onset and wake up times measured from actigraphy data (Facer-Childs et al., 2020). Across all 5 categories, the difference between the groups was significant (Facer-Childs et al., 2019).

Participants attended three scanning sessions at the local times of 14:00, 20:00, and 08:00 the following day. One ECP (Subject 11) had their 14:00 rs-fMRI scan excluded due to excessive movement.

### 2.2. Data acquisition

A Philips Achieva 3 Tesla MRI scanner with a 32-channel head coil was used to collect the imaging data for all participants. This involved a 5-min T1-weighted scan to create a standard high-resolution anatomical image of the brain (1 mm isotropic voxels) before a 15-min eyes-open resting-state scan was obtained. The scans captured the entire brain using gradient echo echo-planer imaging oriented parallel to the AC-PC line with the following parameters: 450 volumes, TR = 2,000 ms, TE = 35 ms, flip angle = 80°, 3 × 3 × 4 mm^3^ voxels. Respiratory and cardiac fluctuations were monitored using equipment also provided by Philips.

### 2.3. Data preprocessing

Pre-processing was completed using UF^2^C (User-Friendly Functional Connectivity) introduced in de Campos et al. (2016) (RRID:SCR_016550). This is a toolbox written in MATLAB (RRID:SCR_001622), which relies upon SPM12 (Penny et al., 2007) (RRID:SCR_019184) and PhysIO (Kasper et al., 2017), which are needed for physiological noise correction. Preprocessing involved standard steps implemented in SPM12 (reorientation to the anterior commissure as origin, rigid body motion correction, spatial normalization to the MNI-152 template, spatial smoothing with a 6 mm^3^ Gaussian kernel, linear detrending). In addition, physiological noise corrections were performed using RETROICOR within the PhysIO toolbox (third order cardiac, fourth order respiratory, and first order interaction Fourier expansion of cardiac and respiratory phase, heart rate variability and respiratory volume per time). As a result, 18 nuisance regressors were added to preprocessing routines in UF^2^C, as well as average signals for white matter and cerebrospinal fluid and the six movement regressors. Data were high (>0.008 Hz) and low (<0.1 Hz) filtered. Scans with an average framewise displacement (Power et al., 2012, 2014) above 0.5 mm were excluded, resulting in the exclusion of the afternoon scan from one participant.

### 2.4. Functional network construction

The brain was parcellated into 70 functional ROIs previously used in de Campos et al. (2016) and based upon the 90 functional ROIs used by the Stanford Find Lab presented in Shirer et al. (2012). This excluded ROIs in the cerebellum due to incomplete coverage. Information about the ROIs used in the study, including MNI coordinates, is provided in Supplementary Table 1 of Supplementary Section 1. The average timeseries for all voxels within each ROI was then extracted before each timeseries was standardized to mean 0 and standard deviation 1.

FNs were then calculated for each subject using Tikhonov partial correlation. A symmetric weighted adjacency matrix with a size 70 × 70 was created for each scan where edge weights are in the range [−1, 1]. Note that all values in the leading diagonal are set to NaN as self-links are not interpretable under partial correlation. Partial correlation was chosen to reduce the contribution from indirect connections (Marrelec et al., 2006, 2009) as well as its superior performance at replicating ground truth networks considered in simulated data studies (Smith et al., 2011; Wang et al., 2014, 2016). Further, Pervaiz et al. (2020) did an extensive review of partial correlation as well as various regularization techniques, and concluded Tikhonov partial correlation as a recommended method. The regularization technique requires adding a scaled identity matrix to the covariance matrix of each subject before partial correlation is calculated using the inverse of the (regularized) covariance matrix, known as the (Tikhonov) precision matrix. The scalar λ, known as the regularization parameter, was optimized by minimizing the difference between the average precision matrix across all participants and the Tikhonov precision matrix of each individual participant. This optimization was completed by summing over all participants the element-wise difference between the average and individual precision matrix before squaring each element. Since the resulting matrix is symmetric, the square root of the sum of the upper triangular elements was calculated. By considering a range of values of the parameter λ, the choice which minimizes this square root sum is considered optimal. For this study, using data from all three scanning sessions λ = 0.0259 was found to be optimal. The regularization λ = 0.0259 was used for the creation of all Tikhonov partial correlation matrices throughout the paper. Further details on the use of Tikhonov partial correlation, as well as the calculation of the regularization parameter are presented in Supplementary Section 2.

For completeness, after the functional networks were completed graph theoretical analyses and NBS Predict were used and compared to the classifier. Details can be found in the Supplementary Sections 4, 5, respectively.

### 2.5. Network-based statistics

After calculating FNs for all subjects, we used NBS to determine dysconnected networks. The group level difference in edge weights for every edge in the Tikhonov partial correlation functional networks across subjects was determined using a *t*-test under a specific contrast—either ECP > LCP or ECP < LCP. In this case each element—in the 70 × 70 t-statistic matrix—represents the difference in the mean edge weights between the two groups resulting in a symmetric matrix whose elements are the output of a two-sample one-sided *t*-test. The t-statistic matrix was then thresholded with the largest connected component of suprathreshold edges, called a dysconnected network, selected as the subnetwork of interest. Here the dysconnected network is a subnetwork of edges that show the highest difference in FC between the two phenotypes. The significance of a dysconnected network was determined using non-parametric permutation testing (*n* = 5,000) to create a familywise error (FWE) corrected *p*-value. This test compares the intensity (the total weight of the edges) of the connected component to a null distribution of connected component intensities created by randomizing the group to which each participant is assigned. The *p*-value was then calculated as the percentage of random permutations whose intensity is larger than that of the measured dysconnected network. The NBS methodology as originally implemented in Zalesky et al. (2010) is outlined in Supplementary Figure 1.

### 2.6. NBS threshold selection

Thresholding is a key step within the traditional NBS methodology. Different t-statistic threshold choices can have a critical impact on network properties such as the number of nodes and edges, and therefore impact the properties of the dysconnected network. When seeking to label each test subject as an ECP and subsequently an LCP, a t-statistic threshold is required for each labeling. The choice of the t-statistic threshold in the original NBS literature is somewhat arbitrary, with no rigorous method suggested. Here we suggest a process for determining these t-statistic thresholds, which is as follows:

For each individual test subject *m*∈{1, 2, …, 38}, label the test subject an ECP while letting the training set retain their chronotype label (determined by MCTQ, saliva samples and actigraphy data). Using the NBS pipeline calculate the 70 × 70 t-statistic matrix across all subjects using the selected contrast; ECP > LCP or ECP < LCP. Each element represents the difference in the mean edge weights between the two groups of extreme chronotypes when the test subject has been artificially placed in the ECP group. Then set $t_{E}^{m}$ as the highest t-statistic threshold such that the network of suprathreshold edges is connected. This value $t_{E}^{m}$ is known as the percolation threshold. Similarly, repeat the steps above with the test subject *m* labeled an LCP to find the percolation threshold for the LCP labeling, setting $t_{L}^{m}$ to this value.

The importance of setting t-statistic thresholds $t_{E}^{m}$ and $t_{L}^{m}$ as percolation thresholds is directly linked to the creation of minimum connected components (MCCs), which were first presented and fully explained in Vijayalakshmi et al. (2015). Effectively, the MCC is a pragmatic balance between the sparsity and density of a network. Interestingly, Vijayalakshmi et al. (2015) found the MCC to be sensitive to subtle changes in FNs resulting from changes to cognitive load in EEG (electroencephalogram) recordings, which were difficult to detect using other methods.

### 2.7. The classifier

**Figure 1:**
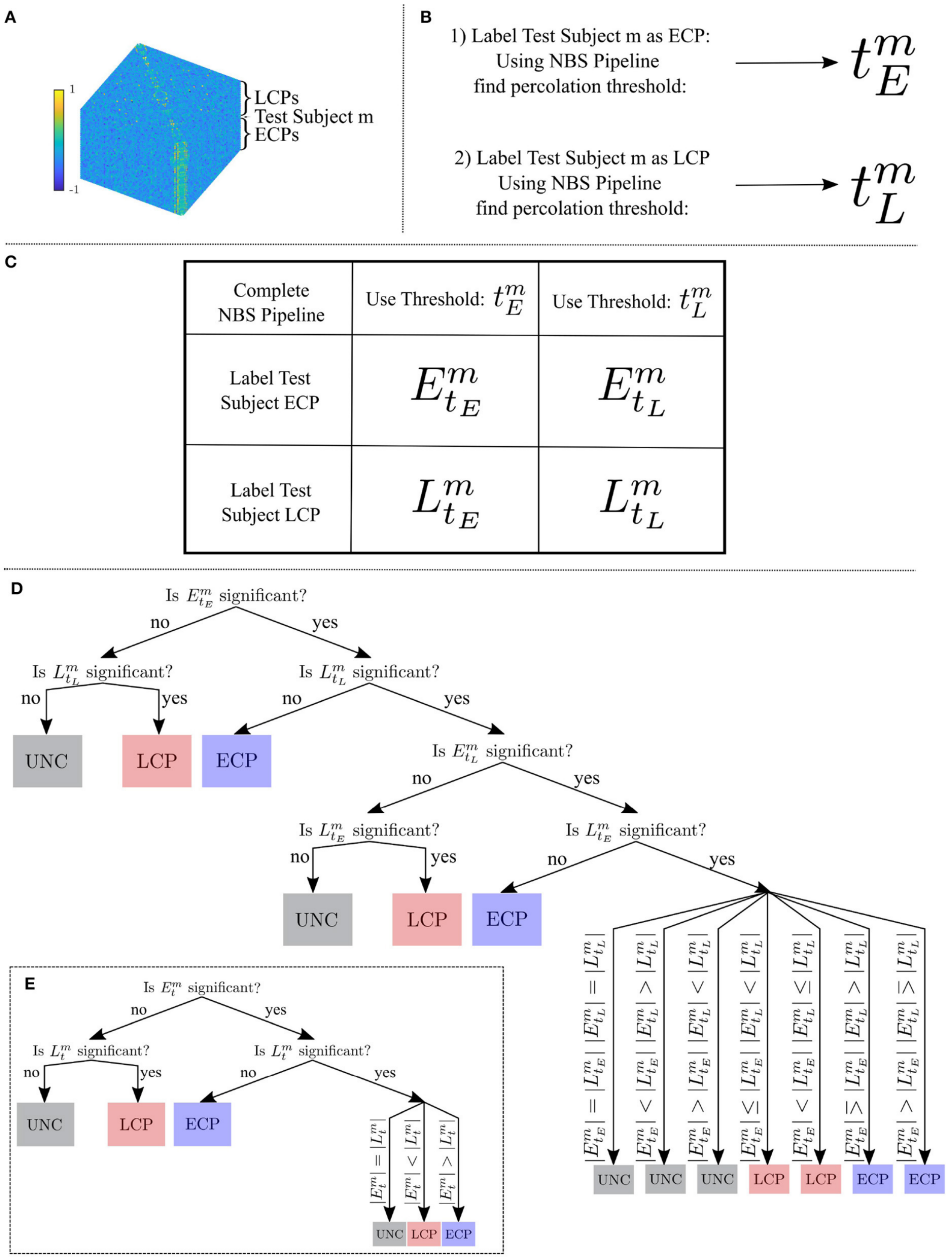
**(A)** All subjects Tikhonov Partial Correlation matrices (70 × 70 × number of subjects) where test subject *m* has a label unknown to the classifier. **(B)** Using the NBS pipeline to find the percolation threshold when the test subject has been labeled an ECP and then an LCP. **(C)** The four dysconnected networks which are created from the two thresholds and two labelings. **(D)** The three steps of the classifier to classify subject *m* as an ECP, an LCP or unclear. **(E)** The reduced classifier needed in the special case, $t_{E}^{m}=t_{L}^{m}=t^{m}$.

To determine whether an individual is an ECP or an LCP, we developed a classifier that considers whether the individual fits best within a family of known ECPs or LCPs. The main steps are summarized in Figure 1.

Let ${CL}_{t}^{m}$ denote the dysconnected network created by assigning subject *m* the chronotype label (*CL*) at t-statistic threshold *t*, calculated using the NBS toolbox (Zalesky et al., 2010). For the two possible chronotype labelings of the test subject and the two t-statistic thresholding values $t_{E}^{m}$ and $t_{L}^{m}$ four subnetworks are created: $E_{t_{E}}^{m}$, $L_{t_{L}}^{m}$, $E_{t_{L}}^{m}$, and $L_{t_{E}}^{m}$. Each subnetwork will have its own FWE-controlled *p*-value where *n* = 5, 000 random permutations of class labels were used to create a null distribution. Here *p* < 0.05 is considered to be significant, and intensity—the sum of the weights—was used when calculating the *p*-value. Intensity was used because the NBS reference manual (Zalesky et al., 2012) suggests calculating the FWE *p*-value using intensity rather than the number of edges is beneficial for detecting subtle (distributed but sparse) effects throughout the network, rather than focal effects within a specific component of the network. From here, the classifier was constructed as follows:

Step one is to consider the significance of $E_{t_{E}}^{m}$ and $L_{t_{L}}^{m}$. If only one of these is significant, the classification of subject *m* is the significant chronotype label *CL*. If neither are significant the classification of subject *m* is unclear. If both are deemed to have a significant size compared to a null distribution then further steps are needed.

Step two is to consider the significance of the two remaining percolation thresholds for each *CL*: $E_{t_{L}}^{m}$ and $L_{t_{E}}^{m}$. In this case, if only one of these is significant, the classification of subject *m* is the significant chronotype label *CL*. If neither are significant then the classification of subject *m* is unclear. If both are deemed to have a significant intensity compared to a null distribution, one further step is required.

Step three is to consider the number of edges |*CL*_*t*_| of the dysconnected networks in t-statistic threshold pairs. For the four significant subnetworks $E_{t_{E}}^{m}$, $L_{t_{L}}^{m}$, $E_{t_{L}}^{m}$, and $L_{t_{E}}^{m}$, the chronotype label with the higher number of edges indicates the label that the classifier will assign to subject *m*—all comparisons are shown in Figure 1. This is based upon the assumption that for any edge, correctly labeling a subject will result in greater separation between the two groups edge weights, which should be reflected in higher t-statistic values. Therefore, at a specific t-statistic threshold, more edges should survive the thresholding step when correctly labeled in contrast to being incorrectly labeled.

Due to the inclusion of the test subject in the classifier, labeled as an ECP or an LCP more advanced techniques such as nested cross-validation cannot be used. Also, note that due to the potential for test subjects to be labeled “unclear”, we define accuracy to be the number of subjects classified correctly divided by the total number of subjects. Similarly, misclassification is the number of subjects given a label of ECP or LCP incorrectly divided by the total number of subjects. Finally, unclear is the number of test subjects given the label unclear divided by the total number of subjects. Hence, accuracy, misclassification and non-classified will add up to 1. In addition, sensitivity can be considered as the number of test subjects classified as ECP divided by the number of ECP subjects and specificity is analogously defined for LCP subjects. Therefore, it should be noted that an unclear classification will not increase accuracy, sensitivity, or specificity. Also, through the *t*-tests the classifier is based on the comparison of two distribution means therefore the imbalance in numbers will not provide a bias toward the classification of the larger group. To clarify that the imbalance in group sizes does not bias classification in this framework, balanced accuracy—the mean of sensitivity and specificity—is also presented within the tables of results.

### 2.8. Varying the t-statistic threshold

A method for selecting the threshold values at which to threshold the t-statistic matrix has already been outlined in Section 2.6. However, the threshold selection process was reliant on the assumption that all ROIs provide differential information. To understand the impact of this assumption we also decided to range the t-statistic thresholding parameter from 0 to 4.5 in increments of 0.01. Therefore, only one t-statistic threshold is selected for both chronotype labelings.

As the t-statistic threshold range extended beyond the percolation threshold some dysconnected networks did not include all ROIs. Therefore, for certain t-statistic thresholds multiple distinct dysconnected networks existed. In this case, we selected the dysconnected network with the smallest FWE *p*-value. When multiple dysconnected networks had the same *p*-value then the dysconnected network with the highest number of edges was selected. If multiple dysconnected networks had the same number of edges then for the purpose of classification they were indistinguishable to the classifier and one was selected arbitrarily.

After selecting the dysconnected networks it can be noted that when $t_{E}^{m}$ and $t_{L}^{m}$ are equal (i.e., ∃ *t*^*m*^∈**R**^+^ s.t. $t_{E}^{m}=t_{L}^{m}=t^{m}$) the classifier can be applied as described in Section 2.7, however to reduce redundancy step one can be removed and step three can be streamlined as shown in Figure 1. For instance, the case ($\left|E_{t_{E}}^{m}|>|L_{t_{E}}^{m}\right|$ and $\left|E_{t_{L}}^{m}|<|L_{t_{L}}^{m}\right|$) is no longer plausible since $E_{t_{E}}^{m}$ and $E_{t_{L}}^{m}$ as well as $L_{t_{E}}^{m}$ and $L_{t_{L}}^{m}$ are identical.

Dysconnected networks for select thresholds were then considered and displayed visually using BrainNet Viewer (Xia et al., 2013) (RRID:SCR_009446).^1^

### 2.9. Investigating the stability of the classifier

When creating a classifier on small datasets, such as the 38 participants available in this study, a sensible step is to validate the classifier on a similar but independent dataset. This allows you to assess whether the classifier is overfitted to the original data and hence its applicability to different datasets. However, with no access to an alternative existing dataset, surrogate datasets were created using the original data. These partial datasets were created by removing one subject in turn from each scanning session; therefore, creating 113 new datasets, across the three scanning sessions (*n* = 37 Afternoon, *n* = 38 Evening, and *n* = 38 Morning).

For each of these new datasets the methodology as outlined above was completed. With accuracy now given as the percentage of correctly labeled subjects calculated from a leave-one-out cross validation analysis on N-1 subjects. Changing the number of subjects will effect the MCC created using NBS both in terms of the size as well as the specific edges included. This will in turn affect the classification of networks in step one and two through the significance of MCCs changing when a subject is removed, as well as the different number of edges affecting step three of the classifier.

Given the overlap between the partial and full datasets we expect the accuracy levels to be consistent. Indeed, any discrepancies in the accuracy when a single subject is removed could indicate potential problems with the classifier or that some characteristics of the participant's data are inherently different and influential.

### 2.10. Varying the threshold for significance

The dysconnected networks that are created using NBS are considered significant if their *p*-value is less than the significance threshold, α = 0.05, such that *p* < 0.05 indicates significance. The threshold of 0.05 is somewhat arbitrary and selected due to the consistent use of this threshold throughout literature. However, the classification pipeline is not solely concerned with the significance of the dysconnected networks, rather whether or not information about chronotype is embedded within them such that classification can occur. Therefore, the threshold for significance was varied in the range [0, 1] in steps of 0.01 to understand its effect on the success of the classifier. This is similar to NBS Predict, however within NBS Predict the *p*-value of the dysconnected network is not even calculated as feature selection is considered irrespective of family-wise error.

The effect of changing the significance threshold was considered for both the original datasets as well as the partial datasets, as mentioned in Section 2.9.

Note that a significance threshold of zero guarantees that every network is non-significant; therefore every classification is unclear due to step one. A non-zero but low significance threshold would result in the majority of subjects being classified due to step one—the significance of the network. As the significance threshold increases step two will be used for classification and finally once the significance threshold is high enough such that all 4 networks (*E*_*t*_*E*__, *E*_*t*_*L*__, *L*_*t*_*E*__, *L*_*t*_*L*__) are all significant then step three—the number of edges—is used. After reaching the significance level such that all four dysconnected networks are considered significant, the accuracy will remain constant for all significance levels higher than this. Therefore, the choice of significance threshold equates to the contribution each step of the classifier makes.

### 2.11. Summary of main steps

A summary of the main steps of the methodological pipeline are given below starting with the creation of functional networks:

1. For each participant a 70 × 70 functional network is calculated where each ROI is a node in the network and Tikhonov Partial Correlation is the functional connectivity measure used to calculate the pairwise connection between ROIs.
2. A single test subject *m* is selected and initially labeled an ECP.

(a) Similar to NBS a 70 × 70 t-statistic matrix is then calculated, such that each element is the result of a *t*-test comparing edge weights for the two groups with Subject *m* considered an ECP.
(b) The t-statistic matrix is then thresholded such that a minimum connected component is created i.e., all 70 ROIs are still connected but a higher threshold will remove at least 1 ROI. The threshold is denoted $t_{E}^{m}$ and the suprathreshold network is called a dysconnected network, denoted $E_{t_{E}}^{m}$ and via the approach in NBS the dysconnected network will have an associated FWE corrected *p*-value.

3. Test subject *m* is then labeled an LCP.

(a) As in NBS a 70 × 70 t-statistic matrix is then calculated, such that each element is the result of a *t*-test comparing edge weights for the two groups with Subject *m* considered an LCP.
(b) The t-statistic matrix is then thresholded such that a minimum connected component is created i.e., all 70 ROIs are still connected but a higher threshold will remove at least 1 ROI. The threshold is denoted $t_{L}^{m}$ and the suprathreshold network is called a dysconnected network, denoted $L_{t_{L}}^{m}$, and via the approach in NBS the dysconnected network will have an associated FWE corrected *p*-value.

4. Step 1: If neither $E_{t_{E}}^{m}$ nor $L_{t_{L}}^{m}$ are significant the test subject is labeled unclear. If $E_{t_{E}}^{m}$ is significant and $L_{t_{L}}^{m}$ is not significant the test subject is labeled an ECP. If $L_{t_{L}}^{m}$ is significant and $E_{t_{E}}^{m}$ is not significant the test subject is labeled an LCP. If both are deemed to have a significant size compared to a null distribution, then further steps are needed.
5. Step 2 if required:

(a) Similarly to above the dysconnected networks $E_{t_{L}}^{m}$ and $L_{t_{E}}^{m}$ are created by labeling test subject *m* an ECP and using threshold $t_{L}^{m}$ and by labeling test subject *m* an LCP and using threshold $t_{E}^{m}$, respectively.
(b) If neither $E_{t_{L}}^{m}$ nor $L_{t_{E}}^{m}$ are significant the test subject is labeled unclear. If $E_{t_{L}}^{m}$ is significant and $L_{t_{E}}^{m}$ is not significant the test subject is labeled an ECP. If $L_{t_{E}}^{m}$ is significant and $E_{t_{L}}^{m}$ is not significant the test subject is labeled an LCP. If both are deemed to have a significant size compared to a null distribution, a further step is needed.

6. Step 3 if required: If all four dysconnected networks are significant then the label is determined by considering the number of edges in each dysconnected network, with more edges taken as an indication of correct labeling, see Figure 1 for all possible comparisons.

## 3. Results

Prior to applying the NBS method, we considered a more traditional connectivity-based approach where graph metrics calculated from the FNs of each subject were compared at the group-level for differences between the two extreme chronotypes. No significant differences were found as shown in Supplementary Section 4, which is consistent with results found independently by Farahani et al. (2021, 2022). In addition, for completeness a comparison to NBS Predict was performed, with the results given in Supplementary Section 5.

### 3.1. Classifier performance

Having selected the t-statistic thresholds $t_{E}^{m}$ and $t_{E}^{m}$ the classifier can be used as presented in Section 2.7. The results for each of the three scans under the two contrasts are presented below. In addition, the classifier labels assigned to each Subject are given in **Table 2**.

#### 3.1.1. Afternoon scanning session

For the contrast ECP > LCP, Subject 22 was mislabeled as an ECP on step one while all other subjects were labeled as unclear by step one, resulting in an accuracy of 0%. For the contrast ECP < LCP, accuracy was 0% with all subjects being classified by step one.

#### 3.1.2. Evening scanning session

For the contrast ECP < LCP, every subject was labeled as unclear by step one, due to $E_{t_{E}}^{m}$ and $L_{t_{L}}^{m}$ being non-significant for all subjects, resulting in an accuracy of 0%.

For the contrast ECP > LCP, 97.37% accuracy was achieved due to only Subject 36 being misclassified as an ECP. The resulting subnetwork when all subjects are correctly labeled (i.e., NBS as presented in Zalesky et al. (2010) is used with t-statistic threshold 1.692) is given in **Figure 4** and its topology is considered in **Table 3**. The breakdown of how many ECPs and LCPs were classified at each step is presented in Table 1.

**Table 1:** The number of ECPs and LCPs that are classified at each step of the classification pipeline is presented in the table as well as the accuracy of the classification.

	**ECP**	**LCP**	**Total**
Step one	10	14	24
Step two	1	1	2
Step three	5	7^†^	12
Accuracy %	100	95.5	97.3

The † denotes Subject 36 who was incorrectly labeled at step three of the classifier.

#### 3.1.3. Morning scanning session

For the contrast ECP > LCP every subject was labeled as unclear by step one, due to $E_{t_{E}}^{m}$ and $L_{t_{L}}^{m}$ being non-significant for all subjects, resulting in an accuracy of 0%. For the contrast ECP < LCP accuracy was 0%, with all subjects being classified by step one.

### 3.2. How the t-statistic threshold impacts classifier performance

As can be seen from Table 2, the performance of the classifier was strongly dependent on the time of day that the scan was taken, with accuracy differing greatly between the Evening and other scanning sessions. Since the accuracy of the classifier for the Afternoon and Morning scans was restricted by dysconnected networks being non-significant at step one of the classifier, when the percolation threshold was used, we now consider the accuracy of the classifier when selecting a single t-statistic threshold, varying over the range [0, 4.5] in increments of 0.01. This relaxes the requirement of all ROIs contributing to the network when choosing the t-statistic threshold for defining the dysconnected network and allows the classifier to be used within a classification framework of understanding if differential information exists with a larger enough effect for classification to occur and if so how the performance of the classifier varies with the t-statistic threshold. Under these conditions, the classifier is the simplified version presented when $t_{E}^{m}=t_{L}^{m}=t^{m}$ where *t*^*m*^ is preselected.

**Table 2:** The labels assigned by the classifier for the different scanning sessions and contrasts.

**Subject**	**Afternoon** ** ECP < LCP**	**Afternoon** ** ECP > LCP**	**Evening ** ** ECP < LCP**	**Evening ** ** ECP > LCP**	**Morning ** ** ECP < LCP**	**Morning ** ** ECP > LCP**
1	UNC	UNC	UNC	ECP	UNC	UNC
2	UNC	UNC	UNC	ECP	LCP	UNC
3	UNC	UNC	UNC	ECP	UNC	UNC
4	UNC	UNC	UNC	ECP	UNC	UNC
5	LCP	UNC	UNC	ECP	UNC	UNC
6	LCP	UNC	UNC	ECP	UNC	UNC
7	LCP	UNC	UNC	ECP	UNC	UNC
8	UNC	UNC	UNC	ECP	UNC	UNC
9	UNC	UNC	UNC	ECP	UNC	UNC
10	LCP	UNC	UNC	ECP	LCP	UNC
11	NA	NA	UNC	ECP	UNC	UNC
12	UNC	UNC	UNC	ECP	UNC	UNC
13	UNC	UNC	UNC	ECP	LCP	UNC
14	UNC	UNC	UNC	ECP	UNC	UNC
15	UNC	UNC	UNC	ECP	UNC	UNC
16	LCP	UNC	UNC	ECP	UNC	UNC
17	UNC	UNC	UNC	LCP	UNC	UNC
18	UNC	UNC	UNC	LCP	UNC	UNC
19	UNC	UNC	UNC	LCP	ECP	UNC
20	UNC	UNC	UNC	LCP	ECP	UNC
21	UNC	UNC	UNC	LCP	UNC	UNC
22	UNC	ECP	UNC	LCP	UNC	UNC
23	ECP	UNC	UNC	LCP	UNC	UNC
24	UNC	UNC	UNC	LCP	UNC	UNC
25	UNC	UNC	UNC	LCP	UNC	UNC
26	ECP	UNC	UNC	LCP	ECP	UNC
27	UNC	UNC	UNC	LCP	ECP	UNC
28	UNC	UNC	UNC	LCP	UNC	UNC
29	UNC	UNC	UNC	LCP	UNC	UNC
30	UNC	UNC	UNC	LCP	UNC	UNC
31	ECP	UNC	UNC	LCP	UNC	UNC
32	UNC	UNC	UNC	LCP	UNC	UNC
33	UNC	UNC	UNC	LCP	UNC	UNC
34	UNC	UNC	UNC	LCP	UNC	UNC
35	UNC	UNC	UNC	LCP	UNC	UNC
36	ECP	UNC	UNC	ECP	ECP	UNC
37	ECP	UNC	UNC	LCP	ECP	UNC
38	ECP	UNC	UNC	LCP	UNC	UNC
Accuracy(%)	0	0	0	97.7	0	0
Balanced accuracy(%)	0	0	0	100%	0	0
Percentage classified ECP or LCP (%)	29.7	2.7	0	100	23.7	0

Here, Subjects 1–16 are ECPs while 17–38 are LCPs, as labeled using non-imaging data. In addition, accuracy, balanced accuracy and the percentage given an ECP or LCP label are provided.

**Figure 2:**
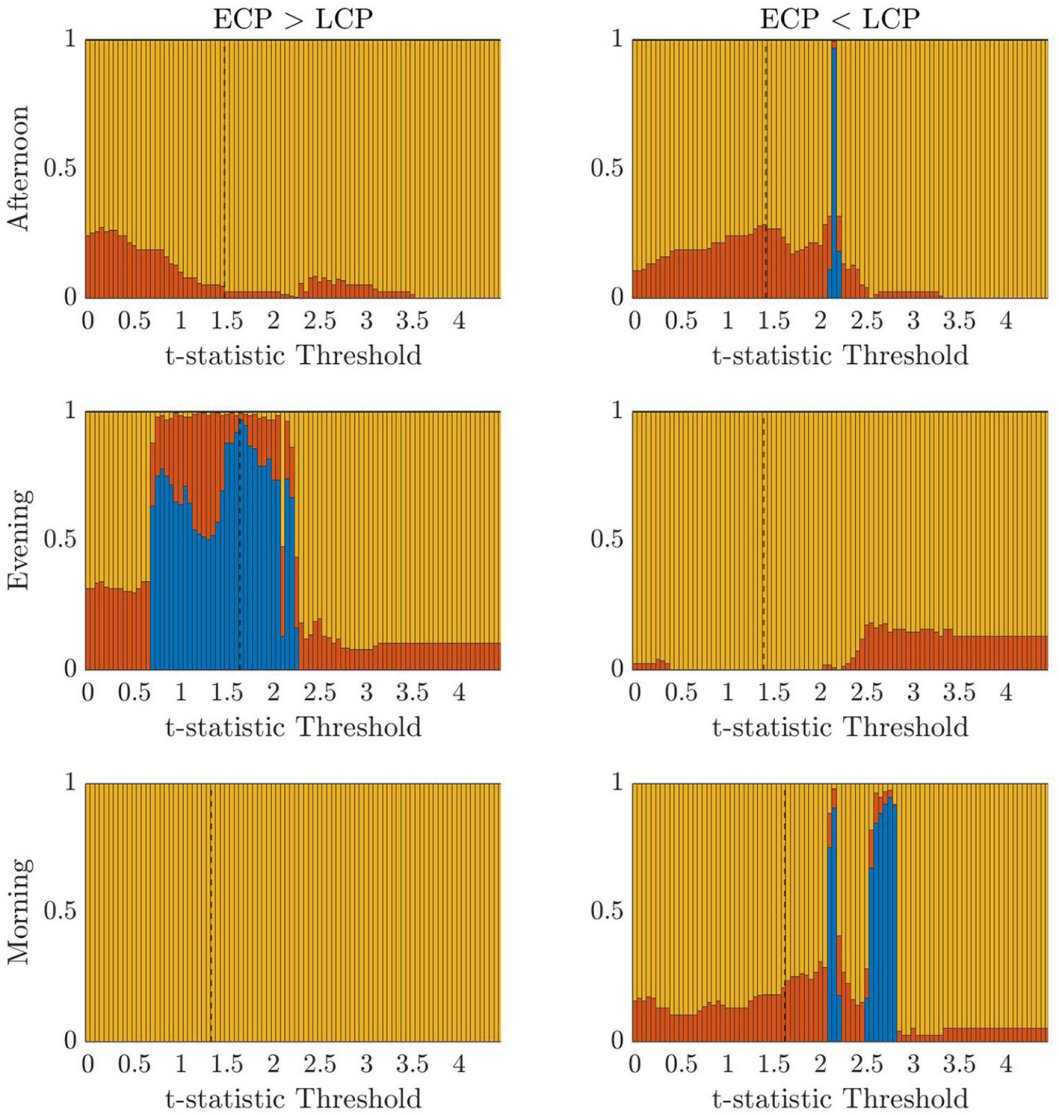
Stacked bar charts showing the fraction of subjects classified correctly, incorrectly and unclear, respectively (blue, red, yellow) when successively selecting the t-statistic threshold from the range [0, 4.5] in increments of 0.01. The dashed lines indicate the percolation threshold for correct labeling as found using Section 2.6.

First, we consider the accuracy values when applying the classifier for the three scanning sessions and two contrasts when varying the t-statistic threshold value. These results are presented in Figure 2. The highest accuracy values for the Afternoon scanning session occur for lower t-statistic thresholds near 2.2 when ECP < LCP (~97%). The contrast ECP > LCP does not result in any non-zero accuracy values. For the Evening scanning session, non-zero accuracy exclusively occurred for the ECP > LCP contrast. For this contrast, non-zero accuracy occurs between 0.71 and 2.28 with accuracy peaking at 97%. In the Morning scanning session, non-zero accuracy was only seen for the ECP < LCP contrast, with t-statistic thresholds near 2.2 and 2.7 with accuracy ~90% for both thresholds.

**Figure 3:**
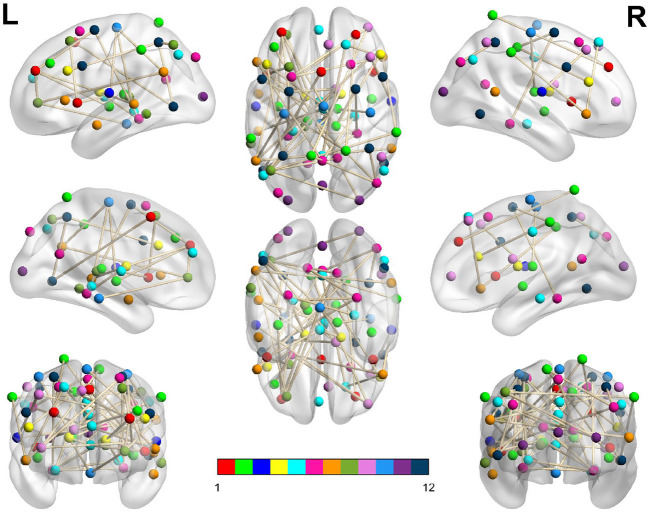
Dysconnected network produced using NBS for the Afternoon scanning session using ECP < LCP and t-statistic threshold 2.19. The top row from left to right are the lateral view of the left hemisphere, top view and the lateral view of the right hemisphere. The middle row from left to right are the medial view of the left hemisphere, bottom view and the medial view of the right hemisphere. The bottom row shows the anterior side on the left and posterior on the right. The interconnected networks (ICNs) 1–12 are given in Supplementary Table 1.

**Figure 4:**
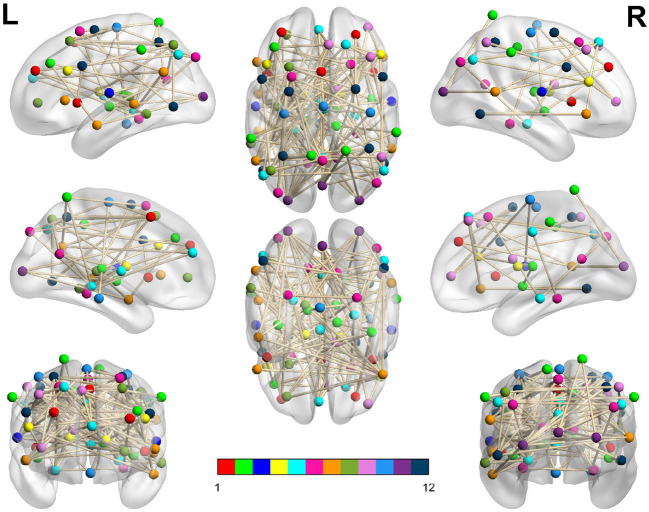
Dysconnected network produced using NBS for the Evening scanning session using ECP > LCP and t-statistic threshold 1.692. Subfigure information is the same as Figure 3.

**Figure 5:**
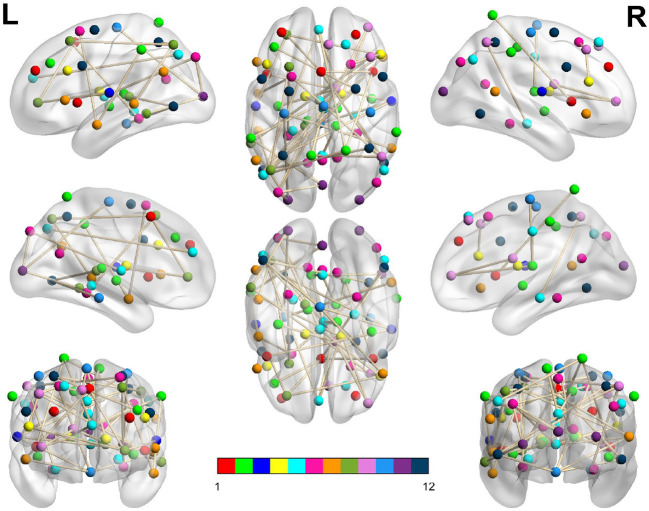
Dysconnected network produced using NBS for the Morning scanning session using ECP < LCP and t-statistic threshold 2.19. Subfigure information is the same as Figure 3.

**Figure 6:**
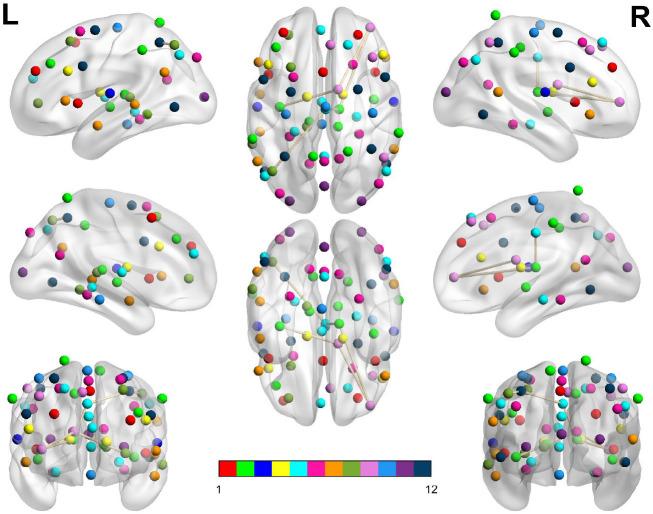
Dysconnected network produced using NBS for the Morning scanning session using ECP < LCP and t-statistic threshold 2.79. Subfigure information is the same as Figure 3.

In Table 3, we display the topologies of four distinct dysconnected networks corresponding to the regions of non-zero accuracy in Figure 2. In addition, Figures 3–6, visualize the four dysconnected networks using glass brains. For the Evening [ECP > LCP] scan the threshold of 1.692 is chosen as it is the percolation threshold when all subjects are correctly labeled as in Section 3.1.2. Since non-zero accuracy did not occur for the Afternoon [ECP > LCP], Evening [ECP < LCP], nor the Morning [ECP > LCP] scanning sessions, no topological features from these scans are considered. Due to the overlap in the t-statistic thresholds that produce high accuracy near at 2.2 in the Morning [ECP < LCP] and Afternoon [ECP < LCP] scanning session, these dysconnected networks at threshold 2.19 were compared for similarity. Notably, only one edge is present in both networks linking nodes 48 (LECN) and 64 (Visuospatial).

**Table 3:** Table showing key topological features of four dysconnected networks.

**Scanning session**		**Threshold**	**Number ** ** of edges**	**Number ** ** of nodes**	**Accuracy (%)**	**Average ** ** node degree**	**Highest node ** **degree (node)**
Afternoon	[ECP < LCP]	2.19	55	53	97.3	1.57 ± 1.22	5 (1)
Evening	[ECP > LCP]	1.69	146	70	97.3	4.17 ± 2.03	10 (60)
Morning	[ECP < LCP]	2.19	53	48	92.1	1.51 ± 1.29	4 (34, 48, 53, 59)
Morning	[ECP < LCP]	2.79	6	7	94.7	0.17 ± 0.54	2 (14, 26, 52, 55, 64)

It is important to note that we do not use this approach to construct a classifier or select a parameter for future datasets, due to the issue of multiple comparisons introduced by varying the threshold over a wide range. The purpose of presenting these results is to provide insight into the somewhat unintuitive differences observed, dependent on time of day, and to understand if differential information does exist in scanning sessions other than the Evening when the MCC assumption is removed.

### 3.3. Investigating the stability of the classifier

So far the results of the classifier for the original datasets have been presented. However, to validate the classifier it is important to understand how the classifier performs on the partial datasets, created by sequentially leaving out participants. Therefore, the results of using the classifier, as presented in Section 2.9, on each of the new datasets created by removing one subject in turn, from both the training set and the test set, are presented below.

#### 3.3.1. Afternoon scanning session

The percentage of subjects who are labeled correct, incorrect, and unclear when a particular subject is removed is shown in Supplementary Tables 5, 6 for the contrasts ECP > LCP and ECP < LCP, respectively.

For the contrast ECP > LCP accuracy was zero for all subjects due to the majority of subjects classified as unclear. When comparing these results to the corresponding column in Table 2 we see a high consistency, as all but one subject was classified as unclear.

For the contrast ECP < LCP there are certain subjects (5, 6, 7, 10, 16, 23, 26, and 38) whose removal has a positive effect on the accuracy of the classification. Indeed, except for Subject 38, the accuracy is higher than random chance (50%) and in some cases the classifier would be considered high performing. For example, the removal of Subject 16 sees accuracy go from 0% up to 81%.

When comparing the results to the corresponding column in Table 2 we see a high correspondence between the incorrectly labeled subjects in Table 2 and those subjects whose removal sees a high accuracy, especially for the ECP cohort.

#### 3.3.2. Evening scanning session

Tables 4, 5 show the percentage of subjects who were labeled correct, incorrect, and unclear when a particular subject was removed, for the contrasts ECP > LCP and ECP < LCP, respectively.

**Table 4:** Evening [ECP > LCP] results for the partial datasets.

**Subject ** ** removed**	**Accuracy**	**Unclear**	**Misclassified**	**Balanced** ** accuracy**
1	0.65	0.14	0.22	0.67
2	0.97	0.03	0	0.98
3	0.78	0.08	0.14	0.79
4	0.78	0.11	0.11	0.80
5	0.95	0.03	0.03	0.94
6	0.84	0.03	0.14	0.85
7	0	0.76	0.24	0
8	0	0.81	0.19	0
9	0	0.73	0.27	0
10	0.73	0.08	0.19	0.75
11	0.65	0.16	0.19	0.65
12	0.70	0.14	0.16	0.71
13	0.81	0.19	0	0.82
14	0.81	0.08	0.11	0.80
15	0.95	0.05	0	09.5
16	0	0.78	0.22	0
17	0.70	0.08	0.22	0.72
18	0.41	0.32	0.27	0.36
19	0	0.78	0.22	0
20	0.86	0.05	0.08	0.87
21	0.95	0	0.05	0.94
22	0.43	0.41	0.16	0.38
23	0.86	0.05	0.08	0.87
24	0.81	0.03	0.16	0.88
25	0	0.81	0.19	0
26	0.84	0.08	0.08	0.85
27	0.68	0.05	0.27	0.68
28	0.59	0.16	0.24	0.60
29	0.92	0.03	0.05	0.92
30	0.43	0.38	0.19	0.38
31	0.86	0.03	0.11	0.87
32	0	0.78	0.22	0
33	0.76	0.11	0.14	0.76
34	0	0.81	0.19	0
35	0.86	0.05	0.08	0.87
36	0.76	0.19	0.05	0.76
37	0.62	0.19	0.19	0.61
38	0.81	0.05	0.14	0.82
Average	0.60	0.25	0.15	0.60

The fraction of correct, unclear, and incorrect classifications when one subject was removed.

**Table 5:** Evening [ECP < LCP] results for the partial datasets.

**Subject** ** removed**	**Accuracy**	**Unclear**	**Misclassified**	**Balanced** ** accuracy**
1	0	1	0	0
2	0	1	0	0
3	0	1	0	0
4	0	1	0	0
5	0	1	0	0
6	0	1	0	0
7	0	1	0	0
8	0	1	0	0
9	0	1	0	0
10	0	1	0	0
11	0	1	0	0
12	0	1	0	0
13	0	1	0	0
14	0	1	0	0
15	0	1	0	0
16	0	1	0	0
17	0	1	0	0
18	0	1	0	0
19	0	1	0	0
20	0	1	0	0
21	0	1	0	0
22	0	1	0	0
23	0	1	0	0
24	0	1	0	0
25	0	1	0	0
26	0	0.97	0.03	0
27	0	1	0	0
28	0	1	0	0
29	0	1	0	0
30	0	1	0	0
31	0	1	0	0
32	0	1	0	0
33	0	1	0	0
34	0	1	0	0
35	0	1	0	0
36	0	1	0	0
37	0	1	0	0
38	0	1	0	0
Average	0	1	0	0

The fraction of correct, unclear, and incorrect classifications when one subject was removed.

For the contrast ECP < LCP no subject was correctly labeled due to almost all subjects being classified as unclear. When comparing these results to the corresponding column in Table 2, we see extremely high consistency.

For the contrast ECP > LCP there are certain subjects (7, 8, 9, 16, 18, 19, 22, 25, 30, 32, and 34) whose removal results in a decrease in accuracy below random chance and far below the 97.3% seen for the full dataset. In addition, the mean accuracy of the other subjects is 79.68%—a reduction from the average of 97.3%. However, it is worth noting that the removal of Subject 36—the only subject incorrectly classified in the original dataset—sees an increase in accuracy from 0 to 76%.

The low or zero accuracy for those 11 subjects is mainly due to the majority of the subjects being classified as unclear. There was no clear link to differences in the non-imaging data (e.g., actigraphy, DLMO, CAR, etc.) that could provide a reason for these subjects having such a clear influence on the classifier's performance.

#### 3.3.3. Morning scanning session

The percentage of subjects who were labeled correct, incorrect, and unclear when a particular subject was removed is shown in Supplementary Tables 7, 8 for the contrasts ECP > LCP and ECP < LCP, respectively.

For the contrast ECP > LCP all subjects were classified as unclear, which is consistent with the corresponding column in Table 2 where all subjects were classified as unclear.

For the contrast ECP < LCP there are certain subjects (3, 10, 13, 20, 26, 27, 29, 34, 36, 38) whose removal has a positive effect on the accuracy of the data. In the case of Subjects 3, 10, 13, 20, 26, 27, and 38 their removal produces accuracy's higher than random chance (50%). For example, the removal of Subject 13 sees accuracy go from 0% up to 76%.

When comparing the results to the corresponding column in Table 2 we see a correspondence between the subjects whose removal sees a high accuracy and incorrectly labeled subjects in Table 2. However, Subjects 3 and 38 were not misclassified in Table 2 whilst Subjects 2, 19, 36, and 37 were misclassified in Section 3.1.3 but their removal had no discernible effect on the classifier.

### 3.4. How the significance threshold impacts classifier performance: partial datasets

Due to the sensitivity of the classifier, both in terms of removing subjects resulting in increased accuracy in the Morning and Afternoon scanning session [ECP < LCP] and the reduction in accuracy in the Evening scanning session [ECP > LCP], the influence of the choice of significance threshold was investigated.

It is worth noting that the initial investigation into the classifier's sensitivity to the removal of subjects focused on the subjects themselves. Therefore, an investigation into the metadata of subjects who were misclassified or whose removal led to high differences in accuracy was undertaken. However, as seen in Supplementary Section 7, the metadata is unable to provide an answer for the classifiers' sensitivity.

**Figure 7:**
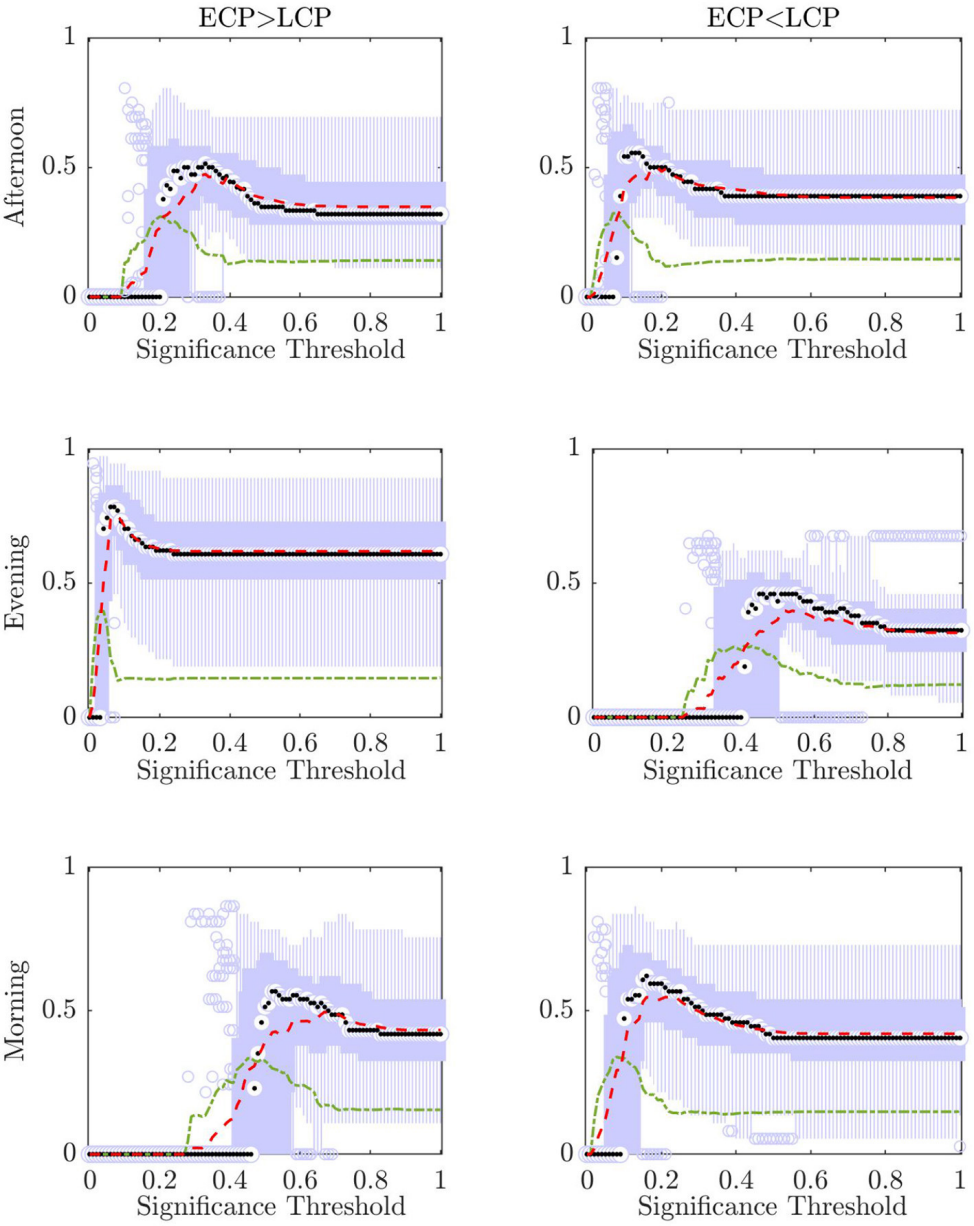
For each value of significance, in the range 0 to 1 in increments of 0.01, a boxplot for the accuracy when removing each subject is shown as the mean accuracy (red, dashed) and the standard deviation (green, dashed, and dotted). The median of the boxplot is given by the black dot while values considered outliers (greater than 2.7 standard deviations away from the mean) are depicted by small blue circles.

**Figure 8:**
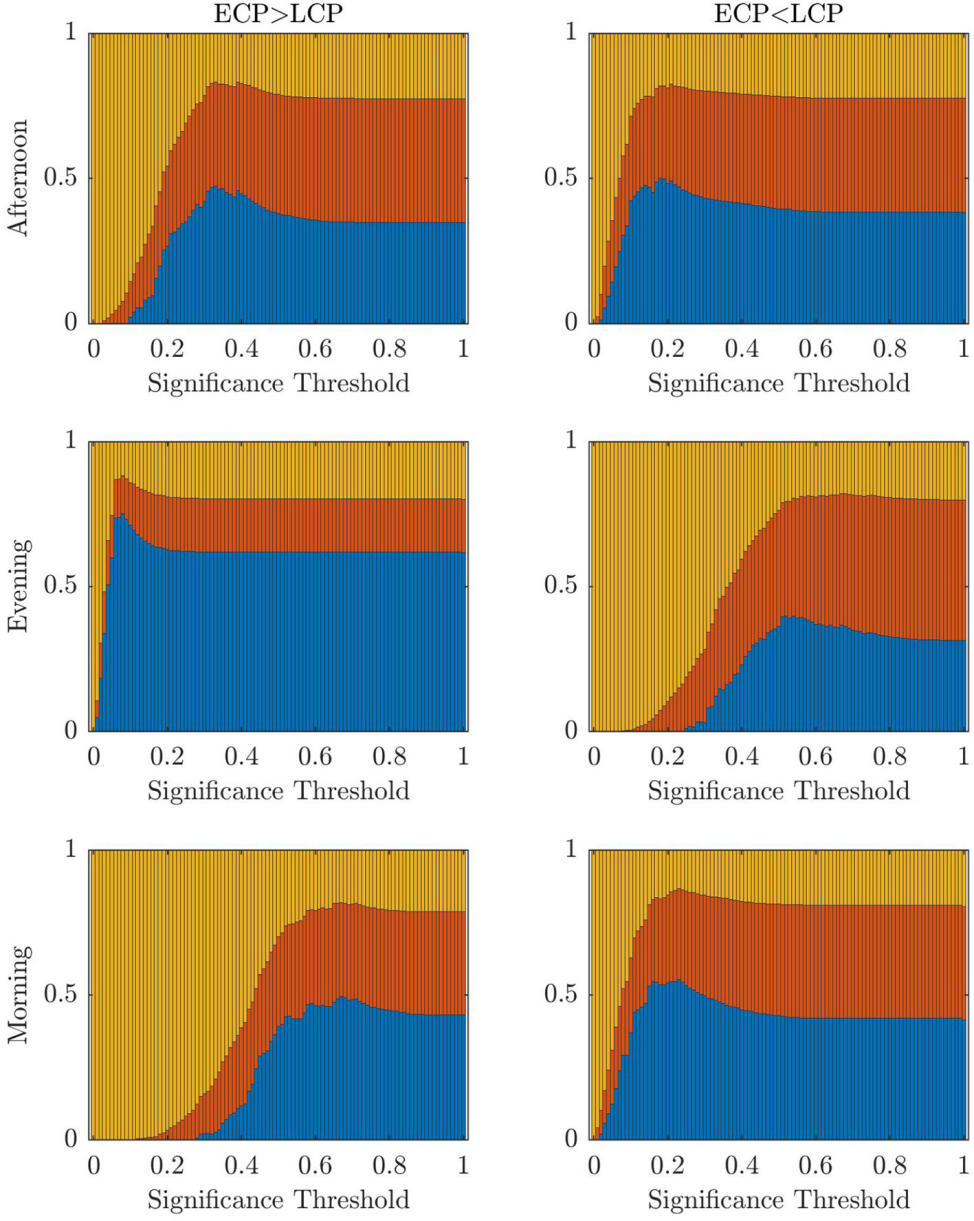
Stacked bar chart showing the mean fraction of subjects classified correctly, incorrectly, and unclear, respectively (blue, red, yellow) when removing one subject in turn and varying across the threshold for significance.

Figure 7 shows boxplots for all of the *n* accuracies when one subject in turn has been removed from the training and test set for all possible values of the significance threshold α. In addition, the mean and standard deviation for each α value are shown. Figure 8 shows the mean value for the percentage of subjects labeled correct, incorrect, and unclear for each α value.

As can be seen from Figure 7 there is an optimum value of α for each of the scanning sessions, selected as the point where the ratio between accuracy and standard deviation is highest.

However, it is only in the Evening [ECP > LCP] scan that the value of α which results in the maximum accuracy matches the value of α which produces the minimum standard deviation. This occurs at α = 0.08 resulting in a mean accuracy of 75.25% showing that there is the possibility to optimize the significance threshold for α and that selecting the traditional significance level of α = 0.05 may be too conservative when there are additional steps of the pipeline that help to prevent misclassification, through the ability to label a test subject unclear.

### 3.5. How the significance threshold impacts classifier performance: original datasets

To understand how changing the significance threshold affects the performance of the classifier when considering the original datasets, the results when varying the significance between [0, 1] in increments of 0.01 are shown below.

**Figure 9:**
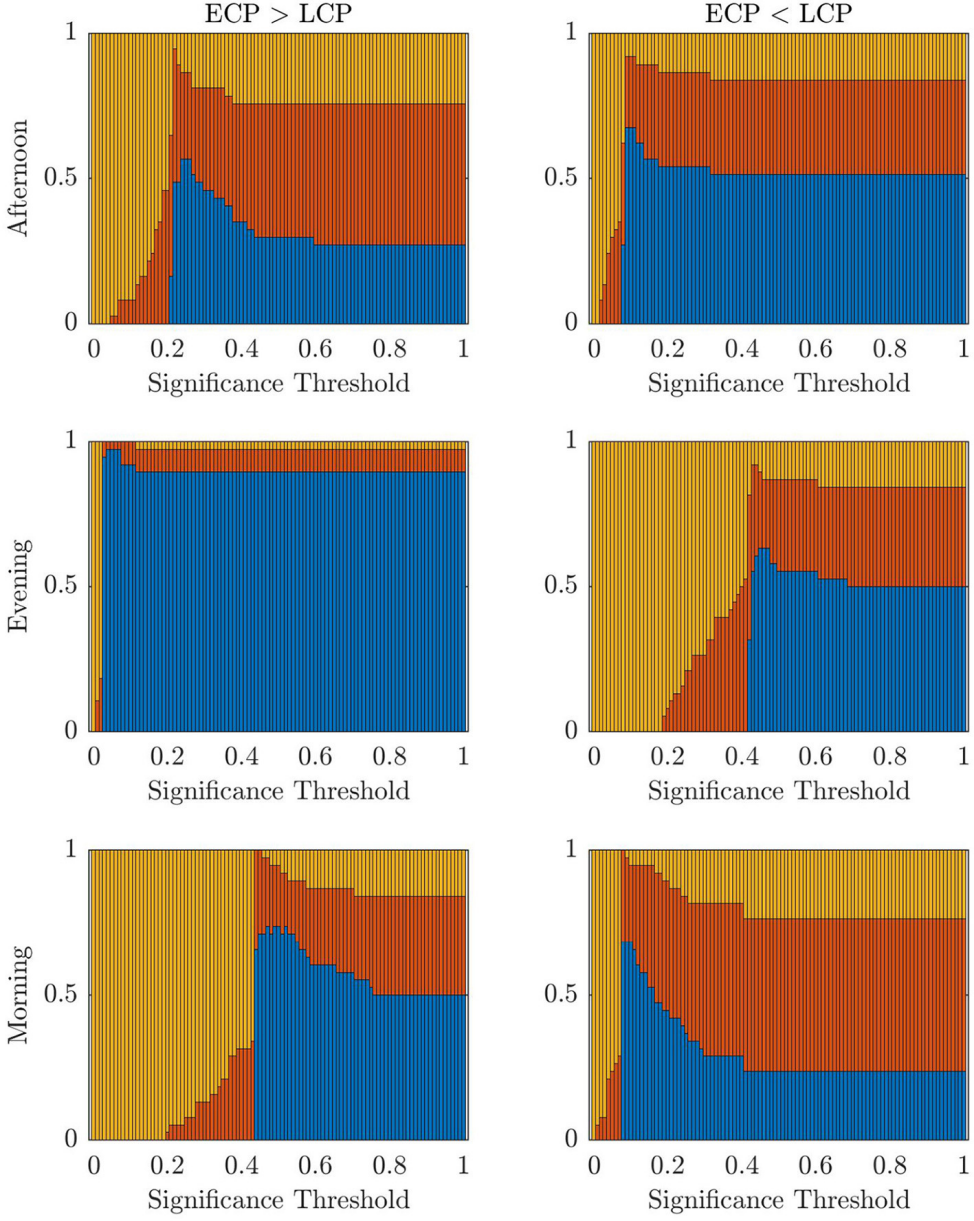
The fraction of subjects classified correctly, incorrectly, and unclear, respectively (blue, red, yellow) when varying across the threshold for significance.

Figure 9 shows a stacked barchart showing the distribution of correct, incorrect, and unclear classification of each possible value of α when ranging from 0 to 1 in increments of 0.01 for the original full datasets. The maximum accuracy of 97% occurs for the Evening [ECP > LCP] scan for α in the range 0.04–0.07. Comparing Figures 7–9, we see similar trends with accuracy increasing with increasing α up to a point specific to each scan and then steadily declining to a plateau once α is high enough. Table 6 shows the values of α for which the maximum accuracy occurs.

**Table 6:** Table showing the values of α which give the highest accuracy with the accuracy values given in brackets.

	**ECP > LCP**	**ECP < LCP**
Afternoon	0.24–0.26 (57%)	0.09–0.11 (68%)
Evening	0.04–0.07 (97%)	0.45–0.47 (63%)
Morning	0.47, 0.49, 0.50 (74%)	0.08–0.1 (68%)

## 4. Discussion

To the best of our knowledge, this is the first study explicitly aiming to classify an individual's chronotype using rs-fMRI data. While previous studies (Horne and Norbury, 2018; Facer-Childs et al., 2019, 2021) have identified differences in functional connectivity associated with chronotype, the question of whether these differences are sufficient to identify a participant's chronotype solely from fMRI data has not been asked. Following the creation of FNs, NBS is used as the base for a classifier. The classifier is innovative through its evaluation of whether an ECP or LCP classification of the test subject leads to a clearer differentiation between the two classes in a group-level comparison.

In addition, the classifier was presented alongside a principled way to select the t-statistic thresholds, a criticism of NBS. This focused on the two percolation thresholds resulting from the two different chronotype labelings a test subject can be assigned. Through concentrating on edges located in dysconnected subnetworks there is evidence that rs-fMRI data does contain enough information to distinguish between ECPs and LCPs. However, this is true only for the Evening [ECP > LCP] scan where we see a high classification accuracy of 97.3% when applying the percolation thresholds.

The high level of accuracy is predominantly due to step one of the classifier, which compares the significance of the MCCs $E_{t_{E}}^{m}$ and $L_{t_{L}}^{m}$. This step contributed almost one third of the correct classifications, as seen in Table 1. Since the MCC, when all subjects are correctly labeled, covers all 70 ROIs with only 146 edges (Table 3), it suggests that the key differences between the FNs of extreme chronotype are sparse and distributed during the evening. This indicates that any influences of chronotype and time of day are impacting the brain relatively globally, which is plausible given that they are driven by systemic circadian and sleep drives. This work and others (Facer-Childs et al., 2019, 2021) suggest that the impact of chronotype on functional networks is relatively subtle, at least within the cortical regions that provide the majority of the data. It would be reasonable to assume that a classifier based on a more focused investigation of for example the suprachiasmatic nucleus of the hypothalamus as the mammalian primary circadian pacemaker would be more sensitive to chronotype differences. However, its small size generally prevents a very specific investigation of its function (Schmidt et al., 2009; Vimal et al., 2009). For the Evening [ECP > LCP] contrast the primary resting state networks were all involved, while the node with the highest degree was in the visual cortex (Supplementary Table 1). Some diurnal variation in visual cortex responsiveness has previously been observed (Vimal et al., 2009). However, regional variation in the sensitivity of cortical and subcortical areas to chronotype and time of day remains an area in need of further investigation.

This may explain why neither the seed-based approach in Fafrowicz et al. (2019), nor the graph metric approach in Farahani et al. (2021) and Farahani et al. (2022) could find significant differences between extreme chronotypes. Since the effect of chronotype is subtle and seen only through the group level comparison of specific edges using a contrast to compare between ECPs and LCPs, this would be lost in many graph metric approaches, which average over many nodes or the entire network reducing the focus on key edges. On the other hand, seed-based approaches that rely on contrasts may identify differences (Facer-Childs et al., 2019, 2021), but the focus on specific seeds could limit the ability to identify the distributed effect seen in the Evening [ECP > LCP] scan.

Meanwhile, the five results of 0% accuracy for the chosen thresholds raises questions about why the classifier's performance is so optimized for the Evening scan and why scans at other time points seem to have no ability to differentiate between ECPs and LCPs. Some variation in the accuracy of the classifier is to be expected because there are known diurnal variations in the graph metrics calculated from FNs in the morning when compared to the evening. Indeed, in Farahani et al. (2021) and Farahani et al. (2022) when ECPs and LCPs were pooled into one group Farahani et al. found significant time of day differences between the morning and evening scanning sessions in small-worldness, assortativity and network synchronization for certain density based thresholds. Therefore, it is likely that the variation in accuracy throughout the day is reflecting dynamic changes in the FNs of extreme chronotypes. Also, Fafrowicz et al. (2019) found significant time of day effects when seeding in multiple areas of the brain. In addition, chronotype is known to affect behavioral outcomes in the two groups differently over the course of the day. For instance, Facer-Childs et al. (2019) found that patterns of subjective sleepiness as measured using KSS for the two extreme chronotypes have an inverse relationship therefore the underlying network dynamics associated with such changes is likely to be reflected in times where the FNs of the two groups show stark changes and times of more similarity.

A key assumption in the presented classifier pipeline is that all ROIs provide valuable information and the effect of chronotype is therefore distributed throughout the brain. However, due to the poor performance across the different scanning session this assumption seems unsuitable for the Morning and Afternoon scans. Previous studies using the same data (Facer-Childs et al., 2019, 2021) have suggested that the differences in FC associated with chronotype and time of day can be restricted to individual nodes, or even sub-portions of individual nodes. The optimal way to define a node (and related issues such as the optimal number of nodes to use etc.) therefore remains an issue and its impact on the classifier requires further research (Song et al., 2016; Korhonen et al., 2017, 2021).

The choice of the t-statistic threshold was investigated to understand how the restriction of the MCC assumption resulted in 0% accuracy in the other 5 cases. In addition, we wanted to understand if the conclusion from Table 2 that the rs-FNs of extreme chronotypes only contain differentiable information in the Evening is true or the result of the MCC assumption. Therefore, the classifier was then used within a classification framework to understand if differential information exists. Figure 2 shows that when the threshold selection process is relaxed it is possible at all three times of the day—under one contrast—to find a t-statistic threshold that results in a highly accurate classifier for distinguishing between ECPs and LCPs. This expands the results presented in Section 3.1, which focus on a specific threshold. However, we restricted our recommendations to cases where a principled approach could be taken to defining the threshold, rather than highlighting situations where good classification accuracy could be achieved with an arbitrary threshold. Clearly, the high classification accuracy resulting from the classification framework supports the view that chronotype classification can be undertaken with rs-fMRI data, although further work is needed to develop the statistical methods to achieve this without the use of arbitrary thresholds or issues of multiple comparisons.

Despite the problem of statistical significance arising from multiple comparisons, Figure 2 does offer some insights into the effect of chronotype over the day, within this dataset despite the inability to determine parameter choices for future datasets. For instance, the contrast resulting in high accuracy, when varying the t-statistic threshold, changes over the course of the day. Simplistically, this generally follows the pattern of high classification accuracy occurring when the chronotype with increased tiredness, as measured using KSS (Facer-Childs et al., 2019), has a higher FC in the contrast. Hence, the contrast ECP > LCP performs better in the evening when ECPs are likely to be more tired than LCPs. Similar logic follows for the Morning scan, when forcing LCPs to awaken before their natural sleep pattern for an 08:00 scanning session will result in increased tiredness for that cohort. Finally, the contrast ECP < LCP produces non-zero accuracy in the Afternoon. However, the range of t-statistic thresholds, which produce non-zero accuracy, is smaller in the Afternoon compared to the Morning. This could be associated to the greater similarity in KSS scores in the two groups at this time. This may suggest that the classification is driven by tiredness (or Process S within the two process model (Borbély and Achermann, 1999) rather than chronotype (related to Process C) *per se*. Within a real-world setting, and not only in relation to rs-fMRI data, differentiating the impacts of sleep homeostasis and circadian drive is difficult. Future studies using laboratory based constant routine or forced desynchrony protocols (Duffy and Dijk, 2002; Kyriacou and Hastings, 2010) could help to understand how sleep drive and circadian phase are differentially manifested in rs-fMRI data and brain networks more broadly.

Furthermore, the t-statistic thresholds producing high accuracy could provide an insight into how chronotype affects the brain throughout the day. Indeed the size of the dysconnected networks producing high accuracy is markedly different throughout the day. The large range of t-statistic thresholds in the Evening start at 0.71, representing a dense distributed effect, while the highest peak in accuracy near 1.7 suggests a sparse but distributed effect. In contrast, in the Afternoon and Morning the high t-statistic thresholds near 2.2 suggest a focal effect concentrated on specific subsets of the brain's FN. Finally, the second peak in the Morning near 2.8 is focused on only 7 nodes and therefore represents a highly spatially specific impact on the brain. More detailed investigation of the spatial distribution of network changes as a function of time of day and chronotype would help to develop these ideas and provide a more specific understanding of how the brain in impacted.

The change in the range of t-statistic thresholds that result in high accuracy explains why the classifier only saw results of 97.3% for Evening [ECP > LCP] scan, and 0% for the other scans and contrasts. This is because it is the only combination where the percolation threshold when all subjects are correctly labeled, shown by the dashed line in Figure 2, falls directly within the range of t-statistic thresholds that consistently sees non-zero accuracy. For the other five combinations this is not the case. This indicates that the assumption of connectedness, the focus on MCCs and the conventional choice of α = 0.05, is suitable for the Evening [ECP > LCP] scan, while a different combination of parameters needs to be used to optimize accuracy for the other scans. Given our results and the discussion above, other approaches could be developed to extract the information needed to provide good classification accuracy in the Morning and Afternoon. Therefore, our work would suggest that differential information is present in the data and a time of day effect is seen not only through the change in contrast, which produces high accuracy, but the size of the dysconnected network. As this conclusion is based on an analysis with a large number of comparisons we suggest that the clustering of the thresholds which produce high accuracy results provides support for this claim. Indeed the high accuracy clusters around specific thresholds, which may indicate a real range of maximal difference, beyond random effect. This is because if the high accuracy was merely due to chance from testing enough thresholds then the distribution of highly accurate thresholds would not be clustered together but would be more uniformly distributed across the t-statistic thresholds and contrasts.

In addition, the stability of the classifier was investigated, through the creation of additional surrogate datasets using a leave-one-out approach. This approach was taken in the absence of an independent dataset for validation, however it should be noted that while offering some insight into the stability such surrogate datasets cannot truly recreate the level of variability that would be seen in a fully independent dataset. It is clear from the results across all 3 scanning session, as seen in Supplementary Tables 5–8 and Tables 4, 5, that the classifier is highly sensitive to the removal or inclusion of certain subjects. In the case of the Afternoon and Morning scanning sessions the removal of certain subjects led to a considerable increase in accuracy, while in the Evening scanning session subject removal has the opposite effect, reducing accuracy, aside from Subject 36.

We see that when comparing the results from the partial datasets to the original datasets there is a correspondence between improved accuracy when incorrectly labeled subjects in Table 2 are removed. This may indicate that these subjects have properties closer to the other chronotype, and hence why their removal improves the classifier. Similarly, we might hypothesize that the near-zero accuracy resulting from the removal of subjects in the Evening scanning session could be because they have properties which clearly identify them as their phenotype and hence their removal reduces the accuracy of the classifier. However, this hypothesis was not supported by differences in the metadata as shown in Supplementary Section 7. Similarly, the metadata offers no insight into why, for example, Subject 36 was misclassified in the Evening [ECP > LCP] scan. Further work is needed to understand the subtleties of the links between resting brain function measured with fMRI and more established markers of chronotype.

Furthermore, it was observed that a large proportion of the subjects were classified as unclear due to non-significant networks occurring at the traditional 0.05 significance level when the number of subjects was reduced by one. To investigate if differentiable information is present when using higher significance thresholds an investigation into the significance threshold was also completed.

The accuracy of the classifier as well as the standard deviation of the *n* different dataset's accuracy for each significance threshold are presented in Figure 7. An optimal choice of significance threshold could be selected for each scanning session and contrast to maximize the accuracy across the *n* datasets and minimize the standard deviation. This relationship is the clearest for the Evening scanning session and indeed using α = 0.08 shows the improved ability to differentiate chronotypes in the Evening scanning session. For the other scanning sessions and contrasts this relationship is not as distinct, but an optimal ratio between these two factors could be located. Directly relating the parameter choice for significance to the classifier's stability offers a solution for how to improve the classifier's performance in future datasets.

When comparing the optimal significance thresholds seen in Figure 7 for the partial datasets, to the optimal values in Table 6 for the original datasets, we clearly see that α = 0.05 is too conservative for *n*−1 subjects compared to *n* subjects. One possible explanation for this is that the number of subjects is quite low and reducing the size further removes important information. Indeed, the reduction in the optimum value for α decreases for the Evening [ECP > LCP] for 38 subjects (0.04–0.07) compared to when 1 subject has been removed (0.08), indicating that as the number of subjects increases, the stability of the classifier increases. However, it is reasonable to assume this trend will not continue indefinitely and that there will be an optimum number of subjects in the training set such that they provide enough information for classification, while also allowing the test subject to have enough influence that changing their label will have an effect on the t-statistic. This highlights that the classifier is reliant on the fact that changing the label of one subject will lead to a detectable impact on the t-statistics, while also having a large enough training set to ensure there is enough distinction between the two cohorts. This approach is optimized to smaller datasets, where mislabeling one subject will have a greater influence. If the number of subjects increased sufficiently (*n* → ∞) it is reasonable to assume the dysconnected networks produced will be the same irrespective of the labeling of the test subject. At that stage this classifier would be redundant and NBS could be used in its traditional form.

For both the partial datasets in Figure 8 and the original datasets in Figure 9, we see the optimal values of the significance threshold follow a time of day effect similar to that observed in Figure 2. Indeed, we clearly see high differences in the optimal value of α for the different contrasts in the Evening and Morning scan with the optimal significance threshold range being lower when the contrast aligns with the group who are experiencing increased tiredness as measured using KSS (Facer-Childs et al., 2019). In the Afternoon, while there is a difference in the optimal parameter choice between the two contrasts in agreement with KSS, the difference between the optimum α value in the two contrasts is smaller, which again could be associated to both groups having a more similar state of tiredness.

This pattern matches studies where excitability, as measured through transcranial magnetic stimulation, is higher when participants are more tired (Huber et al., 2012; Ly et al., 2016). In addition, Petkov et al. (2014) showed that the propensity of people with epilepsy to transition into a seizure state is greater for networks with a higher mean degree, which are therefore considered more excitable. If we view higher FC as a proxy for larger mean degree then the pattern of scanning session, contrast and accuracy would be linked to excitability and consequently tiredness of the two chronotype groups. This suggestion remains to be investigated in more detail, potentially with studies involving explicit sleep deprivation.

Overall, we have provided evidence that when using this classification framework that differentiable information exists in the rs-FNs of extreme chronotypes and this changes over the day. However, to support the use of the classifier as presented in Section 3.1 additional research would be required. For instance, this study is limited by the small sample size, therefore future larger independent datasets would be required in order to validate the conclusions about the time of day effects and to ensure the accuracy of the Evening [ECP>LCP] scan is not over-optimistic. However, larger levels of recruitment may be hindered due to the multiple scanning sessions and the extreme chronotypes required by the protocol. In addition, due to the nature of the classifier considering one test subject at a time and determining if an ECP or LCP label is most suited for this subject, the classifier naturally uses a leave-one-out cross-validation approach. However, it should be noted that a leave-one-out approach can result in high variability and over-fitting may occur.

In addition, optimizing the parameters of the classifier, which include two thresholding choices, should be the aim of future research. Both the value at which to threshold the t-statistic matrix and the threshold for significance must be selected. In both cases the selection of this value is somewhat arbitrary, and typically determined by convention. By varying over these thresholds it becomes clear that there is differentiable and important information present in the Afternoon and Morning scanning sessions and that optimizing these parameters will improve the accuracy and importantly the sensitivity of the classifier to new datasets. Further research is needed to understand how a rigorous selection process or different underlying assumptions could result in selecting objective thresholds that optimize accuracy for the Morning or Afternoon scanning session. Such research may also develop new ways in which fMRI can be used in the study of chronotype as well as lead to greater insight into the impact of chronotypes on brain FNs. This study also motivates the future use of other methods for quantifying brain function to investigate human chronotype. For instance, assessing the pipelines' suitability for use with EEG data would be a natural extension, especially since MCCs were originally shown to be useful at detecting subtle effects on FNs from EEG recordings (Vijayalakshmi et al., 2015). This may result in the Morning and Afternoon scans having non-zero accuracy for the percolation thresholds, offer other interesting insights or simply increase the practicality of recording sessions in relation to cost and location. However, compared to fMRI, EEG has limited spatial sampling of the brain, and a lack of sensitivity to deep brain structures, which are known to be important to sleep and circadian regulation.

Furthermore, this study allowed subjects to sleep using their preferred schedule for 2 weeks prior to the scans. It is currently unknown if the differentiation and accuracy seen at the three scanning times would stay the same if LCPs were constrained to a more traditional work schedule or if high accuracy would occur at a different time of the day. It seems likely that with the additional sleep pressure associated with conforming to the societal day, LCPs would be more easily differentiable from ECPs.

In conclusion, we have shown that extreme early and late chronotypes have differentiable information in their rs-fMRI data that can be used to classify them. Indeed, this classifier demonstrates that two groups of participants whose differences are relatively subtle (i.e., not based on a clinical diagnosis) can be differentiated using rs-fMRI, when traditional seed-based and connectivity-based methods have struggled.

Through this study we have proposed a classifier and investigated its sensitivity and robustness to changes in parameters and the training set. In an ideal scenario the removal of individual subjects would have a negligible effect on the classification accuracy. However, we found the accuracy of the classifier to be strongly dependent on individual participants, although these participants did not appear to be unusual based on the physiological and behavioral data we had available. We found conditions under which this could be mitigated by altering a combination of the threshold for the t-statistic, significance threshold for the dysconnected network and contrast being used. For future chronotype datasets it is recommended to use a contrast which reflects the tiredness of the two groups at the time of the scan, while issues around the optimal thresholds for significance and t-statistic thresholds remain to be clarified for scans other than the evening.

## Data availability statement

The raw data supporting the conclusions of this article will be made available by the authors, without undue reservation.

## Ethics statement

The studies involving human participants were reviewed and approved by the Research Ethics Committee at the University of Birmingham. The patients/participants provided their written informed consent to participate in this study.

## Author contributions

SM, LJ, JT, AB, and EF-C: conception and design. EF-C and AB: data collection. SM, BC, LJ, WW, JT, and AB: data analysis and interpretation. All authors: drafting and revising the manuscript. All authors contributed to the article and approved the submitted version.

## Footnotes

^1^ http://www.nitrc.org/projects/bnv
