# Supplementary material for: Classification of human chronotype based on fMRI network-based statistics
Source: Front Neurosci. 2023 Jun 5;17:1147219. doi: 10.3389/fnins.2023.1147219 (PMC10277557; doi:10.3389/fnins.2023.1147219)
Supplement: Supplementary file 1 [file Data_Sheet_1.PDF]

Supplementary Material

The code used to create Tikhonov Partial Correlation Matrices as well as the classifier is available online at the github repository `https://github.com/sophie-l-mason/NBS_classifier_code`.

1 ROI INFORMATION

Table S1. Table giving the nodes, ICNs (interconnected networks) and MNI Coordinates used for 70 functional connectivity ROIS. In addition, the node degree resulting from the largest connected component for correct labelling for each ROI using the Evening scanning session [ECP>LCP]

Node	ICN Index	ICN	MNI Coordinates	Node Degree	Node	ICN Index	ICN	MNI Coordinates	Node Degree
1	1	Anti Salience	[-33 45 28]	4	36	6	Ventral DMN	[0 -59 59]	4
2	1	Anti Salience	[-45 11 2]	5	37	6	Ventral DMN	[23 19 53]	4
3	1	Anti Salience	[-1 13 51]	5	38	6	Ventral DMN	[28 -37 -13]	2
4	1	Anti Salience	[27 43 33]	3	39	6	Ventral DMN	[44 -77 37]	5
5	1	Anti Salience	[41 13 5]	1	40	7	Language	[-53 21 4]	2
6	2	Post Saliance	[-39 33 34]	6	41	7	Language	[-52 -5 -16]	4
7	2	Post Saliance	[-58 -41 45]	3	42	7	Language	[-52 -35 1]	1
8	2	Post Saliance	[-9 -55 67]	3	43	7	Language	[-54 -59 27]	8
9	2	Post Saliance	[11 -31 49]	1	44	7	Language	[49 25 -5]	7
10	2	Post Saliance	[19 -51 75]	2	45	7	Language	[56 -49 13]	7
11	2	Post Saliance	[60 -36 45]	2	46	8	LECN	[-27 17 52]	7
12	2	Post Saliance	[-12 -27 9]	2	47	8	LECN	[-29 43 2]	2
13	2	Post Saliance	[-36 -15 -1]	4	48	8	LECN	[-42 -67 49]	6
14	2	Post Saliance	[12 -17 13]	4	49	8	LECN	[-58 -45 -7]	3
15	2	Post Saliance	[40 -9 -3]	5	50	8	LECN	[-14 -31 7]	4
16	3	Auditory	[-54 -15 10]	6	51	9	RECN	[39 25 47]	8
17	3	Auditory	[56 -11 13]	2	52	9	RECN	[37 49 5]	2
18	4	Basal Ganglia	[15 -5 13]	2	53	9	RECN	[48 -56 53]	2
19	4	Basal Ganglia	[-14 -7 11]	3	54	9	RECN	[5 35 50]	5
20	4	Basal Ganglia	[-45 19 28]	2	55	9	RECN	[11 -1 19]	3
21	4	Basal Ganglia	[47 27 21]	7	56	10	Sensorimotor	[-31 -21 63]	5
22	5	Dorsal DMN	[-3 47 22]	7	57	10	Sensorimotor	[39 -20 61]	4
23	5	Dorsal DMN	[-48 -71 41]	6	58	10	Sensorimotor	[1 -15 67]	4
24	5	Dorsal DMN	[19 35 53]	5	59	10	Sensorimotor	[-2 -29 -15]	2
25	5	Dorsal DMN	[0 -55 31]	6	60	11	Visual	[-28 -91 7]	10
26	5	Dorsal DMN	[1 -17 41]	3	61	11	Visual	[30 -91 13]	5
27	5	Dorsal DMN	[50 -67 39]	6	62	11	Visual	[0 -79 17]	6
28	5	Dorsal DMN	[-2 -11 7]	1	63	12	Visuospatial	[-27 -3 61]	4
29	5	Dorsal DMN	[-24 -35 -5]	6	64	12	Visuospatial	[-32 -55 51]	5
30	5	Dorsal DMN	[26 -23 -13]	3	65	12	Visuospatial	[-49 7 32]	5
31	6	Ventral DMN	[-12 -61 21]	7	66	12	Visuospatial	[-48 -67 -1]	4
32	6	Ventral DMN	[-25 9 60]	5	67	12	Visuospatial	[27 -1 61]	6
33	6	Ventral DMN	[-28 -39 -11]	3	68	12	Visuospatial	[38 -49 53]	4
34	6	Ventral DMN	[-36 -85 39]	3	69	12	Visuospatial	[49 9 33]	2
35	6	Ventral DMN	[12 -57 19]	1	70	12	Visuospatial	[50 -61 -5]	3

2 DERIVATION OF TIKHONOV PARTIAL CORRELATION MATRIX

Calculation of Tikhonov partial correlation is given below, extended from an outline provided in Pervaiz et al. (2020).

Let $x^m_{i,j} \in \mathbb{R}^{t_p \times n}$ be the i, j^{th} component of data matrix $X^{(m)}$ for Subject m for a rs-fMRI scan with t_p time points and n ROIs. Then for the mth participant an emperical covariance matrix, $\Sigma^{(m)} \in \mathbb{R}^{n \times n}$, can be calculated using,

$$\Sigma^{(m)} = \frac{1}{t_p}\left(X^{(m)} - \bar{X}^{(m)}\right)'\left(X^{(m)} - \bar{X}^{(m)}\right),$$

where $'$ denotes matrix transpose and $\bar{X}^{(m)}$ is the matrix created from vertically stacking the temporal average for each ROI t_p times,

$$\bar{X}^{(m)} = \begin{pmatrix} \bar{x}_1^{(m)} & \bar{x}_2^{(m)} & \cdots & \bar{x}_n^{(m)} \\ \bar{x}_1^{(m)} & \bar{x}_2^{(m)} & \cdots & \bar{x}_n^{(m)} \\ \vdots & \vdots & \ddots & \vdots \\ \bar{x}_1^{(m)} & \bar{x}_2^{(m)} & \cdots & \bar{x}_n^{(m)} \end{pmatrix}; \qquad \bar{x}_j^{(m)} = \frac{1}{t_p}\sum_{i=1}^{t_p} x_{i,j}.$$

Now the Tikhonov covariance matrix is given by,

$$\Sigma_\lambda^{(m)} = \Sigma^{(m)} + \lambda I, \tag{S1}$$

where $I \in \mathbb{R}^{n \times n}$ is an identity matrix. The positive constant λ controls the strength of the regularisation while the matrix I acts as the target matrix during the inversion process Kuismin and Sillanpää (2017). Therefore, $\lambda = 0$ will result in partial correlation whilst higher values of λ will increasingly force off-diagonal elements closer to 0 and diagonal elements closer to 1. The Tikhonov precision matrix, $\Omega_\lambda^{(m)} \in \mathbb{R}^{n \times n}$, with elements $\Omega_{\lambda_{i,j}}^{(m)}$ or to simply notation $\omega_{i,j}^{(m)}$, can then be calculated as,

$$\Omega_\lambda^{(m)} = \left(\Sigma_\lambda^{(m)}\right)^{-1} = \omega^{(m)},$$

using Cholesky decomposition as explained in Krishnamoorthy and Menon (2013) to computationally calculate the inverse of the Tikhonov covariance matrix using code provided by E. Blake [1]. Finally, the Tikhonov partial correlation matrix (TPCM), can be calculated element wise for $i \neq j$ such that each element is given by,

$$e_{i,j}^{(m)} = \frac{-\omega_{i,j}^{(m)}}{\sqrt{\omega_{i,i}^{(m)}\omega_{j,j}^{(m)}}}.$$

Partial correlation by definition requires two variables to compare whilst controlling for at least one other, therefore $i = j$ and the resulting $e_{i,i} = -1$ is meaningless. Hence, values on the main diagonal of the TPCM have been set to NaN and no self-links can be identified by this method.

The values in the TPCM are the FC between different brain regions and can be used as weights for the strength of statistical connections between different ROIs. If $e_{i,j} = 0$ then under the assumption of fMRI data having Gaussian noise Heras and Margalef (2013) there will be no edge between ROIs i and j.

The selection of the optimal regularisation parameter λ in eq(S1) is based upon minimising an objective function.

[1] https://www.mathworks.com/matlabcentral/fileexchange/34511-fast-and-accurate-symmetric-positive-definite-matrix-inverse-using-cholesky-decomposition

First, let $\bar{\Sigma} \in \mathbb{R}^{n\times n}$ be the average empirical covariance for all N participants calculated by taking the element wise mean across $\Sigma^{(m)}$ for the N participants. Also, let $\bar{\Omega} = (\bar{\Sigma})^{-1} \in \mathbb{R}^{n\times n}$ be the precision matrix associated with the average empirical covariance matrix, with elements $\bar{\Omega}_{i,j}$. A matrix $\Gamma_{\lambda}^{(m)} \in \mathbb{R}^{n\times n}$ with elements, $\Gamma_{\lambda_{i,j}}^{(m)}$, can then be defined using,

$$\Gamma_{\lambda_{i,j}}^{(m)} = \left(\sum_{m=1}^{N}\left(\Omega_{\lambda_{i,j}}^{(m)} - \bar{\Omega}_{i,j}\right)\right)^2 = \left(\sum_{m=1}^{N}\left(\omega_{i,j}^{(m)} - \bar{\Omega}_{i,j}\right)\right)^2 .$$

Each element is calculated by summing the difference between the i, j^{th} element of the average precision matrix and each participant's corresponding λ specified Tikhonov precision matrix element before squaring the result.

Therefore, the optimal value of λ denoted λ_{opt} is the one which minimises the square root of the sum of the upper triangular elements in Γ_{λ} and hence is selected for Tikhonov partial correlation,

$$\lambda_{\text{opt}} = \underset{\lambda}{\operatorname{argmin}}\left(\sqrt{\sum_{i<j}\Gamma_{\lambda_{i,j}}}\right) = \underset{\lambda}{\operatorname{argmin}}\left(f(\lambda)\right). \tag{S2}$$

Potential λ values in the range $[0.0001, 1]$ in steps of 0.0001 are considered. However, for new datasets it would still be important to check the data is standardised, λ_{opt} is unique and that $\lambda_{\text{opt}} \neq \lambda^{\text{upper}}$. The value of $\lambda^{\text{upper}} = 1$ has been artificially set based on the data available however if $\lambda_{\text{opt}} = \lambda^{\text{upper}}$ it would be important to understand the behaviour of $f(\lambda)$, to ensure that the argmin has actually been found. A simple example that highlights the importance of this is that if $f(\lambda)$ is a monotonically decreasing function then a search range of $[0, \lambda^{\text{upper}}]$ would always result in $\lambda_{\text{opt}} = \lambda^{\text{upper}}$. Increasing λ^{upper} would therefore see λ_{opt} change accordingly despite no optimum actually existing.

Note that this provides one value of λ for which to regularise the N participants being considered. In this case one value of λ was optimised across all 113 scanning session across the 3 scanning times.

3 NETWORK-BASED STATISTICS PIPELINE

The pipeline for NBS represented in Figure S1.

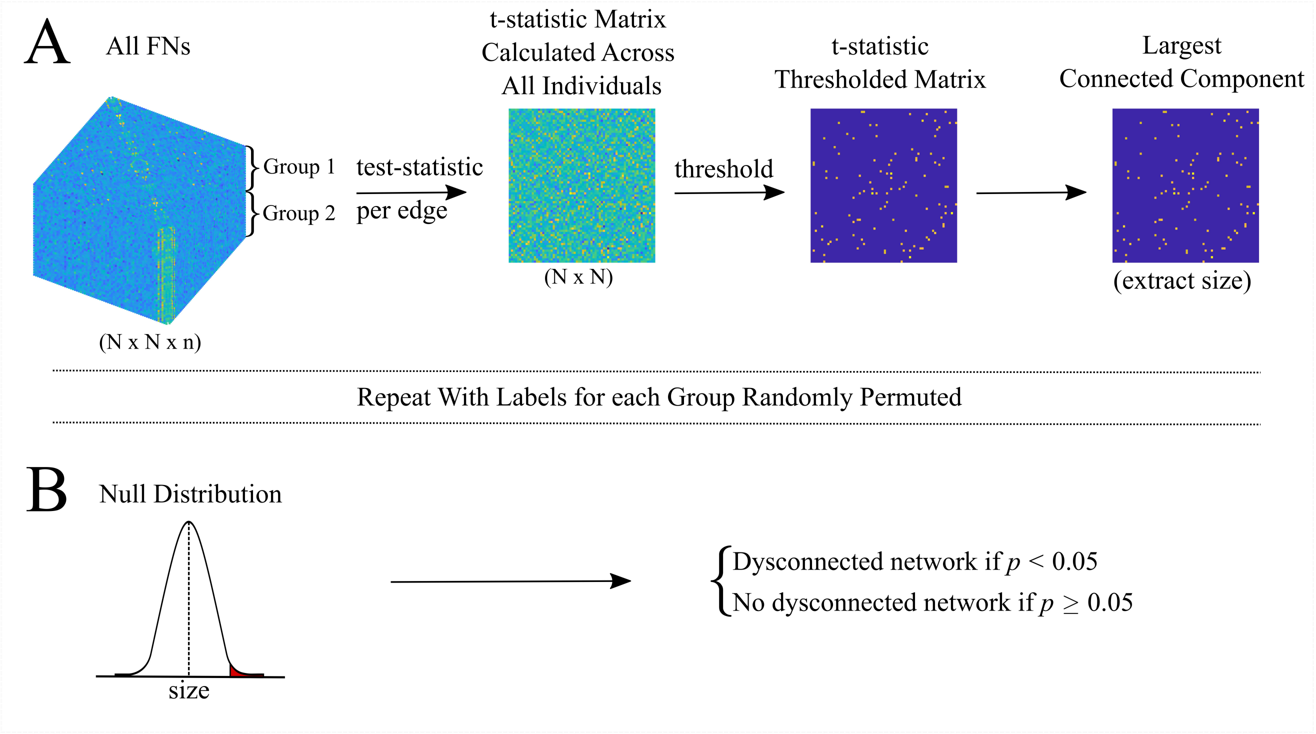

Figure S1. **(A)** The stacked FNs ($N \times N$) of all n subjects with labels assigning them to group 1 (ECP) or group 2 (LCP), from which a t-statistic matrix can be calculated across all subjects per edge. The t-statistic matrix is then thresholded and the largest connected component as well as its size is extracted. This process is then repeated with the group labels randomly permuted 5000 times to create a null distribution of the largest component sizes. **(B)** The size of the actual largest connected component is compared to the null distribution to obtain a p-value. When the largest connected component is significant ($p < 0.05$) a significant dysconnected network exists; otherwise no significant dysconnected network is found.

4 TRADITIONAL GRAPH METRIC APPROACH

Initially, group level analysis was considered using a traditional graph metric approach. The FNs, as constructed in Section 2.4 are used for the base of this analysis.

4.1 Thresholding and Binarizing

Within this study the FNs are thresholded and binarized, such that values below the threshold are set to 0 and values above the threshold are set to 1. The threshold for binarizing the edges ranged incrementally in steps of 0.01 between the lower limit of 0 and the upper limit given by the percolation threshold. The minimum percolation threshold across subjects was selected as the maximum threshold to ensure all networks were connected at all thresholds considered. This creates more physiologically accurate networks, as different brain regions are known to be connected both structurally and functionally.

4.2 Graph Metrics

After the networks have been thresholded and binarized graph metrics were calculated for each network. A limited number of graph metrics ranging across local and global measures were used. These are node degree, node strength, betweenness centrality, clustering, local efficiency, global efficiency, characteristic path length, assortativity and small-world index and small-world propensity. The graph metrics were calculated using the freely available Brain Connectivity Toolbox Rubinov and Sporns (2010)(RRID:SCR_004841) as well as Humphries and Gurney (2008) for small-worldness. In the case of local measures the mean across all nodes was considered. It is worth noting these graph metrics align very closely to those used in a concurrent chronotype study completed by Farahani et al. (2021).

4.3 Group-Level Analysis

Finally, a group level analysis was completed by considering if the graph metrics for the two groups are significantly different at a given threshold value. Significance testing between the two groups distributions were completed using permutation testing ($n = 10,000$ permutations). In addition, corrections for multiple comparisons across graph metrics as well as thresholds were completed using both a Bonferroni correction and the less conservative Benjamini-Hochberg correction.

4.4 Results

The distributions for the different graph metrics for each group are given in Figures S4 to S3 for the three scanning sessions when thresholding and binarizing the whole brain network in increments of 0.01 from 0 up to their MCC threshold.

Figures S2 to S4 show that while there are some combinations of threshold, scanning session and graph metric that are significant with $p < 0.05$ this was only when uncorrected. Correcting for multiple comparisons occurring from testing at multiple thresholds as well as using different graph metrics using a Bonferroni or the less conservative Benjamini-Hochberg correction results in no significance. These results are consistent with those found independently in Farahani et al. (2021).

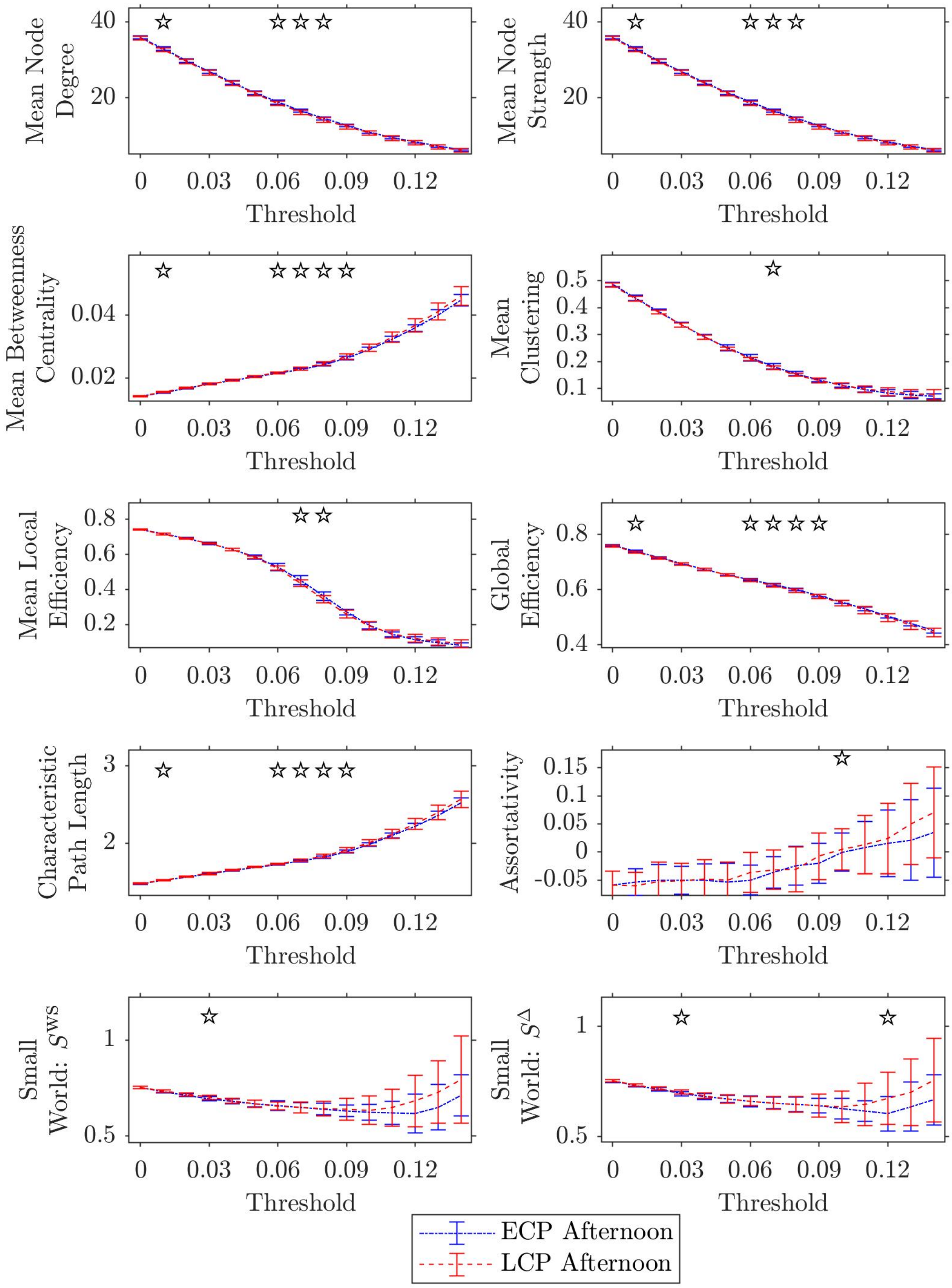

Figure S2. The values for various graph metrics for both ECPs and LCPs within the context of the whole network when binarized for non-negative thresholds increasing in step of 0.01 until the percolation threshold for the afternoon scan. The standard deviation between participants in that group is shown by the error bars. The ☆ indicates where the difference between ECPs and LCPs is significant $p < 0.05$ (uncorrected).

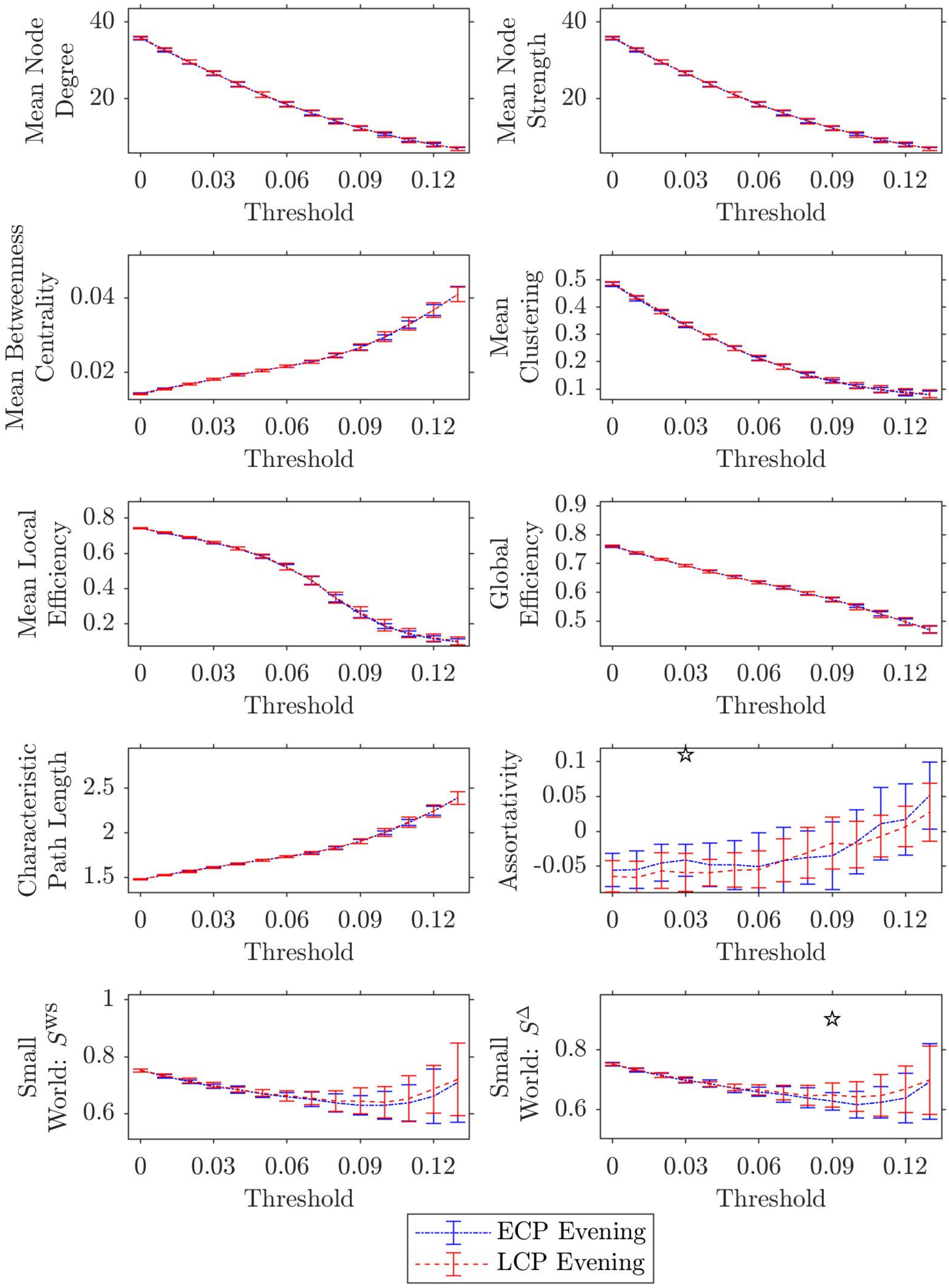

Figure S3. The values for various graph metrics for both ECPs and LCPs within the context of the whole network when binarized for non-negative thresholds increasing in step of 0.01 until the percolation threshold for the evening scan. The standard deviation between participants in that group is shown by the error bars. The ☆ indicates where the difference between ECPs and LCPs is significant $p < 0.05$ (uncorrected).

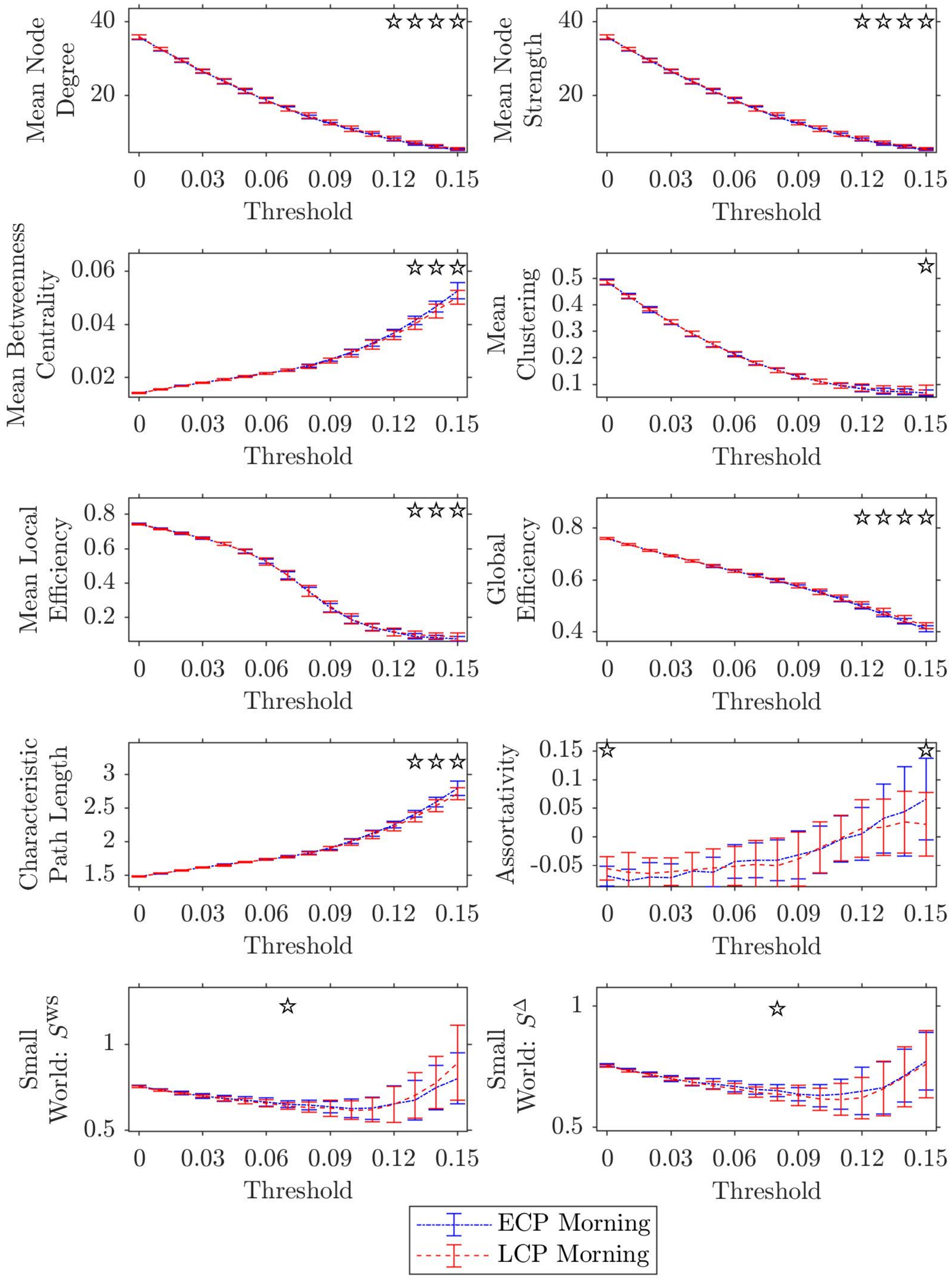

Figure S4. The values for various graph metrics for both ECPs and LCPs within the context of the whole network when binarized for non-negative thresholds increasing in step of 0.01 until the percolation threshold for the morning scan. The standard deviation between participants in that group is shown by the error bars. The ☆ indicates where the difference between ECPs and LCPs is significant $p < 0.05$ (uncorrected).

5 NBS PREDICT

A recent expansion to NBS is NBS Predict Serin et al. (2021), which also aims to classify individuals using parameters from the dysconnected networks. Within the NBS Predict pipeline once again a t-test is calculated for each edge. However, unlike NBS where the t-test is used to create a t-statistic matrix within NBS Predict, the test-statistic matrix contains the associated p-values for each t-test. This p-value matrix is then thresholded at a chosen level of significance, such as 0.05. Therefore only edges with a p-value less than or equal to the threshold remain and the largest connected component, with N edges, is extracted. For each edge with a p-value less than or equal to significance threshold the corresponding functional connectivity values for all participants are used to train a classifier with N features. The classifier model can be logistic regression, Support Vector Machine classification or Linear Discriminant Analysis. Due to the similarity between the aims of the classifier presented in this study and NBS Predict the chronotype dataset was also classified using NBS Predict.

5.1 Results

We present the results of classifying the extreme chronotypes using NBS Predict for each of the three scanning sessions and two contrasts. The result for each classification is given as the accuracy for a LOOCV, where p=0.05. There are three classification types included in NBS Predict, logistic regression, Support Vector Machine classification, and Linear Discriminant Analysis, each of which are considered in Tables S2 - S4 respectively.

Table S2. Table giving the results for each scanning session and contrast when using logistic regression in NBS Predict.

Scanning Session	Contrast	Accuracy	Sensitivity	Specificity	Balanced Accuracy
Afternoon	[ECP > LCP]	0.5946	0	1	0.5000
Afternoon	[ECP < LCP]	0.5946	0	1	0.5000
Evening	[ECP > LCP]	0.5263	0	0.9091	0.4546
Evening	[ECP < LCP]	0.5789	0	1	0.5000
Morning	[ECP > LCP]	0.5526	0	0.9545	0.4773
Morning	[ECP < LCP]	0.5789	0	1	0.5000

Table S3. Table giving the results for each scanning session and contrast when using support vector machine NBS Predict.

Scanning Session	Contrast	Accuracy	Sensitivity	Specificity	Balanced Accuracy
Afternoon	[ECP > LCP]	0.5946	0	1	0.5000
Afternoon	[ECP < LCP]	0.5946	0	1	0.5000
Evening	[ECP > LCP]	0.5000	0	0.8636	0.4318
Evening	[ECP < LCP]	0.5789	0	1	0.5000
Morning	[ECP > LCP]	0.5526	0	0.9545	0.4773
Morning	[ECP < LCP]	0.5789	0	1	0.5000

We see from Table S2 and Table S3, that for two of the models in NBS Predict all of the ECPs are incorrectly labelled as LCPs. Indeed, for at least one contrast in each scanning session all the subjects are labelled as LCPs.

When using Linear Discriminant Analysis in NBS Predict we see a general decrease in the accuracy across 5 of the scanning sessions and contrasts; this is because the classifier has a lower tendency to label

Table S4. Table giving the results for each scanning session and contrast when using Linear Discriminant Analysis NBS Predict.

Scanning Session	Contrast	Accuracy	Sensitivity	Specificity	Balanced Accuracy
Afternoon	[ECP > LCP]	0.5135	0.1333	0.7727	0.4530
Afternoon	[ECP < LCP]	0.7838	0.5333	0.9545	0.7439
Evening	[ECP > LCP]	0.2632	0.0625	0.4091	0.2358
Evening	[ECP < LCP]	0.3421	0.0625	0.5455	0.3040
Morning	[ECP > LCP]	0.3947	0.1875	0.5455	0.3665
Morning	[ECP < LCP]	0.3158	0.1250	0.4545	0.2898

every subject an LCP. However, for Afternoon [ECP < LCP] the accuracy is higher reaching 78% due to 8 ECPs and all but 1 LCP being correctly labelled. This shows that 1 out of the 18 cases has a balanced accuracy greater than 50% and highlights a bias in the labelling of test subjects as LCP across the classifiers, scanning sessions and contrasts. Unfortunately, NBS Predict is unable to provide information on which subjects were incorrectly labelled, preventing comparison at the individual level.

Similar to the results in the Section 3.1 this indicates that a time of day effect is present, as the high accuracy occurred in only one scanning session for one contrast. However, while in Section 3.1 the high accuracy was restricted to Evening [ECP > LCP] when using NBS Predict the high accuracy was restricted to Afternoon [ECP < LCP].

Overall, we see that NBS Predict is highly reliant on the choice of classification method and classification is heavily weighted towards the correct classification of LCPs. Also, if only NBS Predict has been used then this would have given the false impression that neither the Evening nor the Morning scan had any differential information.

6 STABILITY OF THE CLASSIFIER

Table S5. Afternoon [ECP>LCP] results for the partial datasets. The fraction of correct, unclear and incorrect classifications when one subject was removed.

Subject Removed	Accuracy	Unclear	Misclassified	Balanced Accuracy
1	0	0.94	0.06	0
2	0	0.97	0.03	0
3	0	0.97	0.03	0
4	0	0.97	0.03	0
5	0	0.97	0.03	0
6	0	0.97	0.03	0
7	0	0.97	0.03	0
8	0	0.94	0.06	0
9	0	0.94	0.06	0
10	0	0.83	0.17	0
11	NA	NA	NA	NA
12	0	1	0	0
13	0	1	0	0
14	0	1	0	0
15	0	1	0	0
16	0	0.94	0.06	0
17	0	0.94	0.06	0
18	0	1	0	0
19	0	0.94	0.06	0
20	0	0.97	0.03	0
21	0	0.94	0.06	0
22	0	0.92	0.08	0
23	0	0.94	0.06	0
24	0	1	0	0
25	0	1	0	0
26	0	0.97	0.03	0
27	0	0.92	0.08	0
28	0	0.94	0.06	0
29	0	1	0	0
30	0	1	0	0
31	0	1	0	0
32	0	0.94	0.06	0
33	0	0.97	0.03	0
34	0	1	0	0
35	0	1	0	0
36	0	0.97	0.03	0
37	0	1	0	0
38	0	0.97	0.03	0
Average	0	0.97	0.03	0

Table S6. Afternoon [ECP<LCP] results for the partial datasets. The fraction of correct, unclear and incorrect classifications when one subject was removed.

Subject Removed	Accuracy	Unclear	Misclassified	Balanced Accuracy
1	0	0.75	0.25	0
2	0	0.83	0.17	0
3	0	0.81	0.19	0
4	0	0.89	0.11	0
5	0.64	0.03	0.33	0.64
6	0.72	0.11	0.17	0.72
7	0.69	0.03	0.28	0.70
8	0	0.78	0.22	0
9	0	0.78	0.22	0
10	0.78	0.03	0.19	0.77
11	NA	NA	NA	NA
12	0	0.86	0.14	0
13	0	0.81	0.19	0
14	0	0.97	0.03	0
15	0	0.61	0.39	0
16	0.81	0.11	0.08	0.80
17	0	0.92	0.08	0
18	0	0.83	0.17	0
19	0	0.78	0.22	0
20	0	0.75	0.25	0
21	0	0.75	0.25	0
22	0	0.78	0.22	0
23	0.61	0.06	0.33	0.60
24	0	0.72	0.28	0
25	0	0.86	0.14	0
26	0.64	0	0.36	0.63
27	0	0.89	0.11	0
28	0	0.75	0.25	0
29	0	0.78	0.22	0
30	0	0.86	0.14	0
31	0	0.69	0.31	0
32	0	0.94	0.06	0
33	0	0.81	0.19	0
34	0	0.94	0.06	0
35	0	0.72	0.28	0
36	0	0.67	0.33	0
37	0	0.67	0.33	0
38	0.39	0.28	0.33	0.33
Average	0.14	0.64	0.21	0.14

Table S7. Morning [ECP>LCP] results for the partial datasets. The fraction of correct, unclear and incorrect classifications when one subject was removed

Subject Removed	Accuracy	Unclear	Misclassified	Balanced Accuracy
1	0	1	0	0
2	0	1	0	0
3	0	1	0	0
4	0	1	0	0
5	0	1	0	0
6	0	1	0	0
7	0	1	0	0
8	0	1	0	0
9	0	1	0	0
10	0	1	0	0
11	0	1	0	0
12	0	1	0	0
13	0	1	0	0
14	0	1	0	0
15	0	1	0	0
16	0	1	0	0
17	0	1	0	0
18	0	1	0	0
19	0	1	0	0
20	0	1	0	0
21	0	1	0	0
22	0	1	0	0
23	0	1	0	0
24	0	1	0	0
25	0	1	0	0
26	0	1	0	0
27	0	1	0	0
28	0	1	0	0
29	0	1	0	0
30	0	1	0	0
31	0	1	0	0
32	0	1	0	0
33	0	1	0	0
34	0	1	0	0
35	0	1	0	0
36	0	1	0	0
37	0	1	0	0
38	0	1	0	0
Average	0	1	0	0

Table S8. Morning [ECP<LCP] results for the partial datasets. The fraction of correct, unclear and incorrect classifications when one subject was removed.

Subject Removed	Accuracy	Unclear	Misclassified	Balanced Accuracy
1	0	0.95	0.05	0
2	0	0.65	0.35	0
3	0.73	0.03	0.24	0.74
4	0	0.73	0.27	0
5	0	0.81	0.19	0
6	0	0.81	0.19	0
7	0	0.78	0.22	0
8	0	0.76	0.24	0
9	0	0.95	0.05	0
10	0.65	0.05	0.30	0.65
11	0	0.95	0.05	0
12	0	0.89	0.11	0
13	0.78	0.05	0.16	0.81
14	0	0.78	0.22	0
15	0	0.92	0.08	0
16	0	0.84	0.16	0
17	0	0.86	0.14	0
18	0	0.86	0.14	0
19	0	0.78	0.22	0
20	0.62	0.14	0.24	0.62
21	0	0.89	0.10	0
22	0	0.89	0.10	0
23	0	0.76	0.24	0
24	0	0.86	0.14	0
25	0	0.84	0.16	0
26	0.57	0.16	0.27	0.57
27	0.73	0.11	0.16	0.74
28	0	0.86	0.14	0
29	0	0.68	0.32	0
30	0	0.92	0.08	0
31	0	0.89	0.11	0
32	0	0.86	0.14	0
33	0	0.86	0.14	0
34	0	0.76	0.24	0
35	0	0.81	0.19	0
36	0	0.68	0.32	0
37	0	0.73	0.27	0
38	0.65	0.03	0.32	0.65
Average	0.12	0.69	0.19	0.13

7 METADATA

7.1 Subjects Metadata

All the subjects were screened as ECPs or LCPs through a combination of actigraphy data, DLMO and CAR concentrations as well as their mid point of sleep on free days (MSF) when corrected for sleep debt (MSF_{SC}). Therefore, subject metadata was considered to see if this offers an explanation for why the classifier is sensitive to certain subjects.

The metadata does seem to offer some explanation for why the classifier is so sensitive to the removal of certain subjects, especially in the case of ECPs as the MSF and DLMO appear to be lower for the subjects who were incorrectly labelled in the Afternoon and Evening scan. However, when corrected for multiple comparisons using Benjamini-Hochberg test no significance remains.

Therefore, across the three scanning sessions there no significant information to support that the metadata of subjects provides explains the sensitivity of the classifier to a subjects removal especially. This is further supported because, across the scanning sessions there is no consistency in the subjects whose removal has the biggest effect. Indeed, across all 3 scanning sessions the removal of 21 subjects leads to a high difference in the accuracy. Therefore, over half of the subjects cannot be considered outliers according to the metadata or the data would be fundamentally flawed.

REFERENCES

Farahani, F. V., Fafrowicz, M., Karwowski, W., Bohaterewicz, B., Sobczak, A. M., Ceglarek, A., et al. (2021). Identifying diurnal variability of brain connectivity patterns using graph theory. *Brain Sciences* 11, 111. doi:10.3390/brainsci11010111

Heras, O. A. and Margalef, A. G. (2013). *Partial Correlation Network Analysis*. Master's thesis, Department of Mathematics, Universitat Pompeu Fabra. IBE-Final Year Project

Humphries, M. D. and Gurney, K. (2008). Network 'small-world-ness': A quantitative method for determining canonical network equivalence. *PLoS ONE* 3, e0002051. doi:10.1371/journal.pone.0002051

Krishnamoorthy, A. and Menon, D. (2013). Matrix inversion using cholesky decomposition. *Signal Processing: Algorithms, Architectures, Arrangements, and Applications (SPA)* , 70–72

Kuismin, M. O. and Sillanpää, M. J. (2017). Estimation of covariance and precision matrix, network structure, and a view toward systems biology. *WIREs Computational Statistics* 9, e1415. doi:10.1002/wics.1415

Pervaiz, U., Vidaurre, D., Woolrich, M. W., and Smith, S. M. (2020). Optimising network modelling methods for fmri. *NeuroImage* doi:10.1016/j.neuroimage.2020.116604

Rubinov, M. and Sporns, O. (2010). Complex network measures of brain connectivity: Uses and interpretations. *NeuroImage* 52, 1059–1069. doi:10.1016/j.neuroimage.2009.10.003

Serin, E., Zalesky, A., Matory, A., Walter, H., and Kruschwitz, J. D. (2021). Nbs-predict: A prediction-based extension of the network-based statistic. *NeuroImage* 244. doi:2021.118625
